# Whole-genome optical mapping of bone-marrow myeloma cells reveals association of extramedullary multiple myeloma with chromosome 1 abnormalities

**DOI:** 10.1038/s41598-021-93835-z

**Published:** 2021-07-19

**Authors:** Eva Kriegova, Regina Fillerova, Jiri Minarik, Jakub Savara, Jirina Manakova, Anna Petrackova, Martin Dihel, Jana Balcarkova, Petra Krhovska, Tomas Pika, Petr Gajdos, Marek Behalek, Michal Vasinek, Tomas Papajik

**Affiliations:** 1 Department of Immunology, Faculty of Medicine and Dentistry, Palacky University Olomouc and University Hospital Olomouc, Hnevotinska 3, 779 00 Olomouc, Czech Republic; 2 Department of Hemato-Oncology, Faculty of Medicine and Dentistry, Palacky University Olomouc and University Hospital Olomouc, Olomouc, Czech Republic; 3 Department of Computer Science, Faculty of Electrical Engineering and Computer Science, VŠB-Technical University of Ostrava, Ostrava, Czech Republic

**Keywords:** Haematological cancer, Molecular biology

## Abstract

Extramedullary disease (EMM) represents a rare, aggressive and mostly resistant phenotype of multiple myeloma (MM). EMM is frequently associated with high-risk cytogenetics, but their complex genomic architecture is largely unexplored. We used whole-genome optical mapping (Saphyr, Bionano Genomics) to analyse the genomic architecture of CD138+ cells isolated from bone-marrow aspirates from an unselected cohort of newly diagnosed patients with EMM (n = 4) and intramedullary MM (n = 7). Large intrachromosomal rearrangements (> 5 Mbp) within chromosome 1 were detected in all EMM samples. These rearrangements, predominantly deletions with/without inversions, encompassed hundreds of genes and led to changes in the gene copy number on large regions of chromosome 1. Compared with intramedullary MM, EMM was characterised by more deletions (size range of 500 bp–50 kbp) and fewer interchromosomal translocations, and two EMM samples had copy number loss in the 17p13 region. Widespread genomic heterogeneity and novel aberrations in the high-risk *IGH/IGK/IGL*, 8q24 and 13q14 regions were detected in individual patients but were not specific to EMM/MM. Our pilot study revealed an association of chromosome 1 abnormalities in bone marrow myeloma cells with extramedullary progression. Optical mapping showed the potential for refining the complex genomic architecture in MM and its phenotypes.

## Introduction

Multiple myeloma (MM) is a clonal plasma cell proliferative disorder usually limited to a bone marrow (BM) microenvironment. Rarely, patients present with extramedullary disease (EMM), in which myeloma cells spread to other organ systems^1–3^. This aggressive and mostly treatment-resistant sub-entity of MM can either accompany a newly diagnosed disease, occurring at a frequency of 3–18%^4,5^, or develop with disease progression or relapse, with a frequency of 6–20%^4,6^. Currently, little is known about the mechanisms leading to the development of EMM, stroma-independent growth and the survival of myeloma cells at extramedullary sites or the reasons for poor treatment responses. There is growing evidence that genetic factors may contribute to EMM pathogenesis and evolution^1,4,5^.

Genetic studies have shown that high-risk abnormalities, such as 1q21 gain and del(1p32) (detected in > 55% of EMM patients), t(4;14) (~ 52%), *MYC* overexpression (~ 38%), del(17p13) (~ 35%) and del(13q14) (~ 31%), are commonly associated with EMM^1,4,5^. The disruption of the *TP53* gene by del(17p) and/or mutations seems to be a crucial driver of EMM (EMM vs MM: 34.5% vs 11.9%)^7,8^. Mutations in the *RAS*^9^, *KRAS, PIK3CA, ATM* and *NFKB2*^1^ genes have also been associated with the presence of EMM, including *CRBN* mutations leading to treatment resistance^10^. Other important aberrations in EMM include the activating mutations in the NF-κB pathway genes and the homozygous deletion of the genes encoding inhibitors of this pathway^11^. The resulting constitutive activation of NF-κB enhances the expression of adhesion molecules, such as integrin VLA-4, CD-44, P-selectin and numerous chemokines/receptors^6,12^, leading to the migration and stroma-independent growth of myeloma cells^11^. Additional genetic aberrations may occur in patients with extramedullary mass due to clonal evolution^7,13^. However, the complex genetic architecture in MM and EMM is still poorly understood, likely due to its complexity and heterogeneity.

Therefore, we applied novel whole-genome optical mapping to investigate the complex genomic architecture of BM myeloma cells in newly diagnosed MM and EMM patients. This method has an advantage in detecting small and large structural rearrangements as well as complex rearrangements across the whole genome that are undetectable by traditional methods, such as sequencing and cytogenetics^14^. The characterisation of genetic architecture in EMM could significantly contribute to the understanding of EMM pathogenesis with the potential to discover new prognostic and diagnostic biomarkers and improve the outcome of this MM entity. Moreover, a comparison of MM and EMM may help to elucidate genetic events, allowing the dissemination of myeloma cells from BM to blood and distant tissues.

## Materials and methods

### Subject enrolment

BM aspirates were obtained from an unselected cohort of 11 newly diagnosed MM patients with EMM presentation (n = 4; median age: 77 years, min–max: 51–79; M/F: 3/1) and without EMM (MM, n = 7; 75 years, 62–82; 5/2). Patients were diagnosed according to the International Myeloma Working Group criteria^15^. The only criteria for patient enrolment were sampling at diagnosis and a sufficient number of sorted cells to perform all genetic analyses (≥ 2 million myeloma cells). In our patients, all EMM sites were bone related, with two in the thoracic spine and two in the pelvis (one in the iliac bone and one in the acetabulum). Patient’s clinical and demographic data are summarised in Table 1 and Table S1. For all patients, karyotype, FISH (fluorescence in situ hybridization, Table S2), arrayCGH (Table S3) and next-generation sequencing (NGS) for mutations in the *TP53, KRAS, NRAS* and *BRAF* genes (Table S2) were available.

**Table 1:** Basic demographic and clinical characteristics of enrolled MM and EMM patients.

Clinical features	All patients (n = 11)	EMM (n = 4)	MM (n = 7)
Male/female	8/3	3/1	5/2
Age (years), median (min–max)	77 (51–82)	77 (51–79)	75 (62–82)
**ISS staging, n (%)**			
ISS I	5 (45.5)	2 (50.0)	3 (42.9)
ISS II	1 (9.1)	0 (0.0)	1 (14.3)
ISS III	5 (45.5)	2 (50.0)	3 (42.9)
**Durie-Salmon stage, n (%)**			
IA	1 (9.1)	0 (0.0)	1 (14.3)
IIA	4 (36.4)	1 (25.0)	3 (42.9)
IIIA	4 (36.4)	2 (50.0)	2 (28.6)
IB	1 (9.1)	0 (0.0)	1 (14.3)
IIIB	1 (9.1)	1 (25.0)	0 (0.0)
**LC + FLC, n (%)**			
IgG kappa	5 (45.5)	3 (75.0)	2 (28.6)
IgA kappa	4 (36.4)	0 (0.0)	4 (57.1)
IgA lambda	2 (18.2)	1 (25.0)	1 (14.3)
**Cytogenetic analysis**^**a**^**, n (%)**			
t(4;14)	1 (9.1)	0 (0.0)	1 (14.3)
t(11;14)	1 (9.1)	0 (0.0)	1 (14.3)
Gain (1q21)	6 (54.5)	2 (50.0)	4 (57.1)
del(13q14)	4 (36.4)	1 (25.0)	3 (42.8)
del(1p32)	1 (9.1)	1 (25.0)	0 (0.0)
del(17p)	0 (0.0)	0 (0.0)	0 (0.0)
Monosomy	5 (45.5)	2 (50.0)	3 (42.8)
Trisomy	9 (81.8)	4 (100.0)	5 (71.4)
Tetrasomy	5 (45.5)	3 (75.0)	2 (28.6)
**NGS analysis**^b^**, n (%)**			
*TP53*	0 (0.0)	0 (0.0)	0 (0.0)
*KRAS*	2 (18.2)	1 (25.0)	1 (14.3)
*NRAS*	0 (0.0)	0 (0.0)	0 (0.0)
*BRAF*	3 (27.3)	1 (25.0)	2 (28.6)

*ISS* International Staging System, *LC* monoclonal protein's light chain, *FLC* free light chain.

^a^10% positive cut-off level used.

^b^The full coding sequence of the *TP53* gene (exons 2–11, plus 5′ and 3′UTR; NM_000546) and the hotspot regions in *NRAS* (exons 2–4; NM_002524), *KRAS* (exons 2–4; NM_004985) and *BRAF* (exons 11 and 15; NM_004333) were sequenced.

All patients provided written informed consent about the usage of BM for this study, which was performed in accordance with the Helsinki Declaration and approved by the ethics committee of the University Hospital and Palacký University Olomouc.

### Collection of BM aspirates

BM aspirates (2.5–10 ml) were collected in a 5 ml RPMI-1640 medium (Sigma-Aldrich, MO, USA) containing 5000 IU/ml heparin (Zentiva, Prague, Czech Republic). BM mononuclear cells (BMMCs) were collected after red blood cell lysis (155 mM NH_4_Cl, 10 mM KHCO_3_, 0.1 mM Na_2_EDTA, pH 7.3) by centrifugation (1000*g*, 5 min). After washing with phosphate-buffered saline containing 0.5 M EDTA (Sigma-Aldrich) and 2% FBS (Thermo Fisher Scientific, MA, USA), the total count of BMMCs and the infiltration of CD138+ cells were determined by BD FACSCanto II (BD Biosciences, CA, USA). CD138+ plasma cells were enriched using an EasySep Human CD138 positive Selection Kit II (STEMCELL Technologies, Vancouver, Canada), according to the manufacturer’s instructions. The enriched myeloma cells were quantified by BD FACSCanto II (BD Biosciences, CA, USA) using a combination of CD19/CD38/CD45/CD56/CD138 antibodies (BioLegend, CA, USA). After centrifugation (2000*g*, 2 min), dry pellets of 0.6–2.5 million myeloma cells were stored at − 80 °C for further analysis.

### Isolation of high molecular weight DNA, labelling and analysis

Frozen myeloma cell pellets were processed following the Bionano Prep SP Frozen Cell Pellet DNA Isolation Protocol^16^. High molecular weight (HMW) genomic DNA was isolated using the SP Blood and Cell Culture DNA Isolation Kit (Bionano Genomics, CA, USA, #80030), according to the manufacturer’s recommendations. DNA quantification was performed using the Qubit dsDNA BR assay kit (Thermo Fisher Scientific) with a Qubit 2.0 Fluorometer (Thermo Fisher Scientific).

A total of 750–1000 ng of HMW DNA was then labelled using the Bionano Prep Direct Label and Stain DLS DNA Kit (Bionano Genomics, #80005), according to the manufacturer’s protocol^17^. The HMW-labelled DNA (within the recommended range of 8–25 labels/100 kbp) was loaded into the Saphyr Chip (Bionano Genomics, #20319) flow cell at a concentration of 4–12 ng/μl and analysed using a Bionano Saphyr instrument, according to the manufacturer’s instructions^18^, targeting 100–300× human genome coverage by collecting 500–1300 GB of data per sample.

### Data assembly, structural variant calling and the identification of breakpoint regions

**Figure 1:**
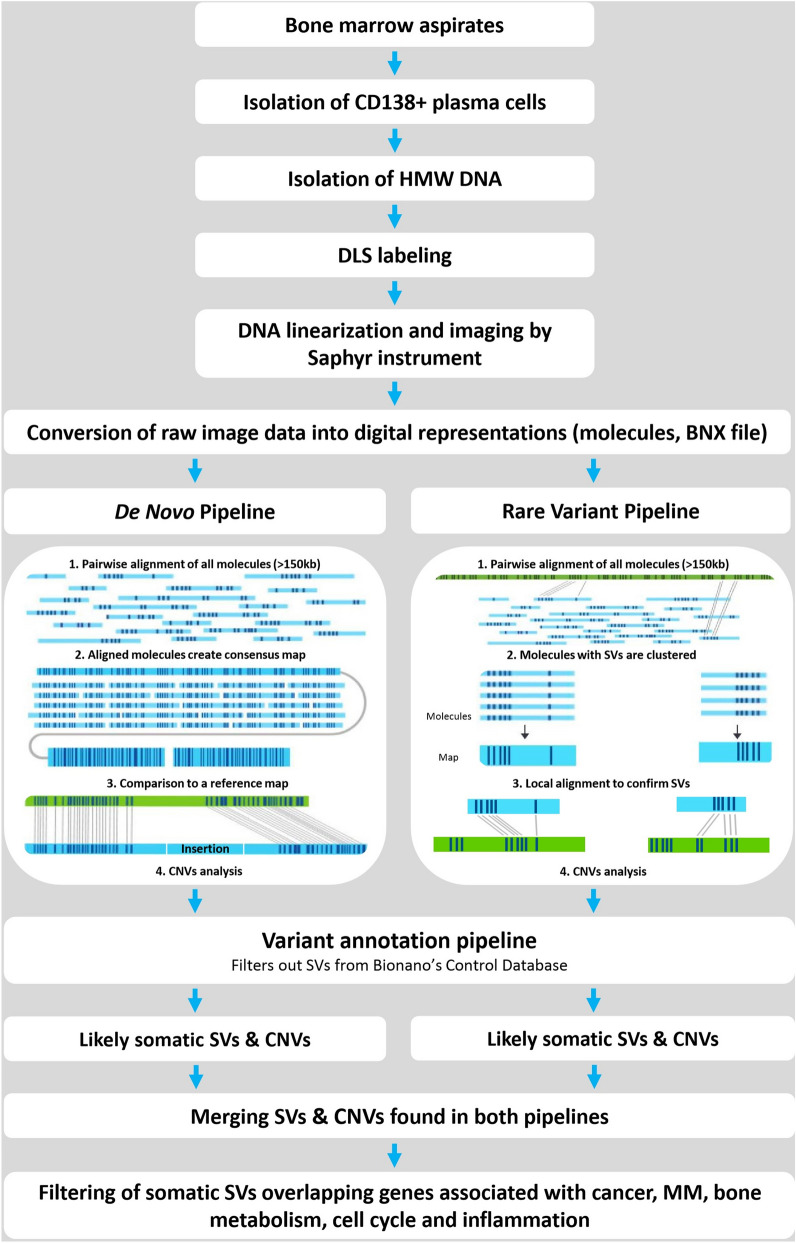
Workflow of optical mapping and bioinformatics pipelines used. HMW DNA is isolated from CD138+ plasma cells of BM aspirates and labelled by DLS chemistry in specific sequences across entire genomes. Labelled DNA is loaded on the chip and linearised and visualised in a Saphyr instrument. Images are converted to BNX molecules. The architecture of the bioinformatics pipeline includes two pipelines (de novo and rare variant), constructing optical genome maps and comparing them with a human reference map (hg38), filtering detected variants for somatic SVs and merging data from both pipelines. The last step enables a comparison of the data with the gene panels created from NCBI gene datasets.

All data were analysed using Bionano Access software (v1.5) containing the Bionano Solve tool (v3.5) and featuring both de novo and rare variant bioinformatics pipelines (Fig. 1), according to the manufacturer’s recommendations^1–21^. Only DNA molecules with a minimum length of 150 kbp were used for bioinformatics analysis along with a minimum of nine labels per molecule.

Briefly, the de novo pipeline’s first assembly of all single molecules was based on the distinct distribution of sequence labels by pairwise alignment. The aligned molecules created consensus maps (contigs) in de novo genome maps, which were compared with the in silico DLE1 labelled human hg38 reference map. This pipeline revealed structural variants (SVs) from 500 bp to tens of Mbp long. In the rare-variant pipeline, all single molecules were pairwise aligned against the hg38 reference assembly; molecules with SVs were clustered, and the obtained maps were locally aligned to the hg38 reference sequence. This pipeline was sensitive enough to detect SVs from 5 kbp to tens of Mbp long at a variant allele frequency (VAF) as low as 5%. SVs were considered subclonal (i.e. low-allele frequency) when VAF was ≤ 25% and clonal (i.e. high-allele frequency) when VAF was > 25%, based on a cut-off value for neutral evolution in MM^11^. Additionally, both pipelines included copy number variation (CNV) analysis to detect the fractional copy number changes and chromosomal aneuploidy events. Specific hg38 masks concealing common structural variation in a human genome, N-base reference gaps and problematic sub-centromeric and sub-telomeric regions were used in both pipelines. To annotate the SV calls that were likely somatic variants, a variant annotation pipeline was applied to filter SVs out of the database of ethnically diverse, mapped control human genomes with no reported disease phenotypes.

In the next step, annotated SVs and CNVs from both pipelines were merged (Fig. 1), including aberrations sized 500 bp–5 Mbp (deletions, insertions, duplications and inversions) as well as inter- and intrachromosomal aberrations larger than 5 Mbp. The intrachromosomal rearrangements with breakpoints at least 5 Mbp apart, e.g. large deletions (supported by copy number loss), insertions (copy number gains) or inversions (no change in CNVs) were called intrachromosomal translocations by the Bionano software (Fig. S1). Only SVs with VAF > 5% and a minimum of ten self-molecules were further analysed in this study. Identified candidate SVs were confirmed by arrayCGH, FISH, breakpoint-specific PCR amplification and/or long-read whole-genome sequencing (TELL-Seq, Universal Sequencing Technology, CA, USA). For a comparison of optical mapping and long-read sequencing data, we developed our own tool, which is available at http://olgen.cz/en/resources^22^.

Finally, the sample-specific SVs were compared with BED masks generated from the NCBI gene database (https://www.ncbi.nlm.nih.gov/gene) for gene panels associated with cancer (created using the keywords cancer, tumour suppressor and oncogene; panel of 10,812 genes), MM (696 genes), bone metabolism (osteolysis, cellular calcium signalling, bone metabolism; 1810 genes), cell cycle (cell signalling, cell division, apoptosis, cell cycle, DNA repair; 9750 genes) and inflammation (inflammation, cell migration, adhesion molecules, cytokine/receptor, chemokine/receptor; 4741 genes).

### NGS mutation assessment

The full coding sequence of the *TP53* gene (exons 2–11, plus 5′ and 3′UTR; NM_000546) and the hotspot regions in *NRAS* (exons 2–4; NM_002524), *KRAS* (exons 2–4; NM_004985) and *BRAF* (exons 11 and 15; NM_004333) were analysed by targeted, ultra-deep NGS, as reported previously^23,24^. Amplicon-based libraries were sequenced as paired ends on MiSeq (2 × 151 bp, Illumina, CA, USA), with a minimum target read depth of 5000×. The detection limit was set up to 1%, and the variants within 1–3% were confirmed by replication.

### Cytogenetic and molecular cytogenetic analysis

After culturing the heparinised BM aspirates in the BM medium (Biological Industries, CN, USA) overnight with colcemid (Gibco, Thermo Fisher Scientific), the samples were processed as reported previously^25^, and at least ten metaphases were karyotyped. A combination of FISH with immunophenotyping, called fluorescence-immunophenotyping and interphase cytogenetics as a tool for investigation of neoplasms (FICTION), was used to assess the cytogenetic abnormalities using the following probes: LSI RB1 (Abbott Molecular, IL, USA), SPEC IGH, SPEC CKS1B/CDKN2C, TP53/c17, CCND1/IGH, FGFR3/IGH (Zytovision, Bremerhaven, Germany), XL MAF/IGH, CCND3/IGH, MAFB/IGH (MetaSystems, Altlussheim, Germany) and centromeric probes for chromosomes 7, 9, 11 and 15 (Cytocell, Cambridge, United Kingdom), as reported previously^25^. ArrayCGH was performed using SurePrint G3 CGH/CGH + SNP 4 × 180 K microarray (Agilent Technologies, CA, USA)^26^.

### Ethics declarations

All patients provided written informed consent about the usage of bone marrow samples for this study, which was performed in accordance with the Helsinki Declaration and approved by the ethics committee of the University Hospital Olomouc and Palacký University Olomouc.

### Consent for publication

This manuscript has been viewed and approved by all authors for publication.

## Results

### Sample analysis by optical mapping

The infiltration of myeloma cells in BM aspirates based on immunophenotyping was highly variable in enrolled patients (3–36%); more than 10% infiltration of plasma cells was found in the BM smears of all enrolled patients. The inter-individual variability in the myeloma cell infiltration may be linked to patchy or site-varied myeloma cell distribution, haemodilution, aspirate pull order, the aggregation of myeloma cells in aspirated BM, myeloma cell immunophenotypes and time-dependent losses of surface markers^23^, as well as disease heterogeneity itself^27^. The infiltration of myeloma cells in all samples after enrichment was > 80% (81–96%). Optical mapping was performed in all enriched samples with the following run parameters: average effective coverage, 154× (min–max: 78–324×); collected data per sample, 699 GB (427–1710 GB); DNA molecule size (N50), 316 kbp (219–446 kbp); label density 17.3 labels per 100 kbp (14.1–22.6); and map rate, 74.4% (41.5–93.3%). The quality control parameters for each sample are summarised in Table S4.

### Detection of SVs and CNVs in myeloma samples

**Figure 2:**
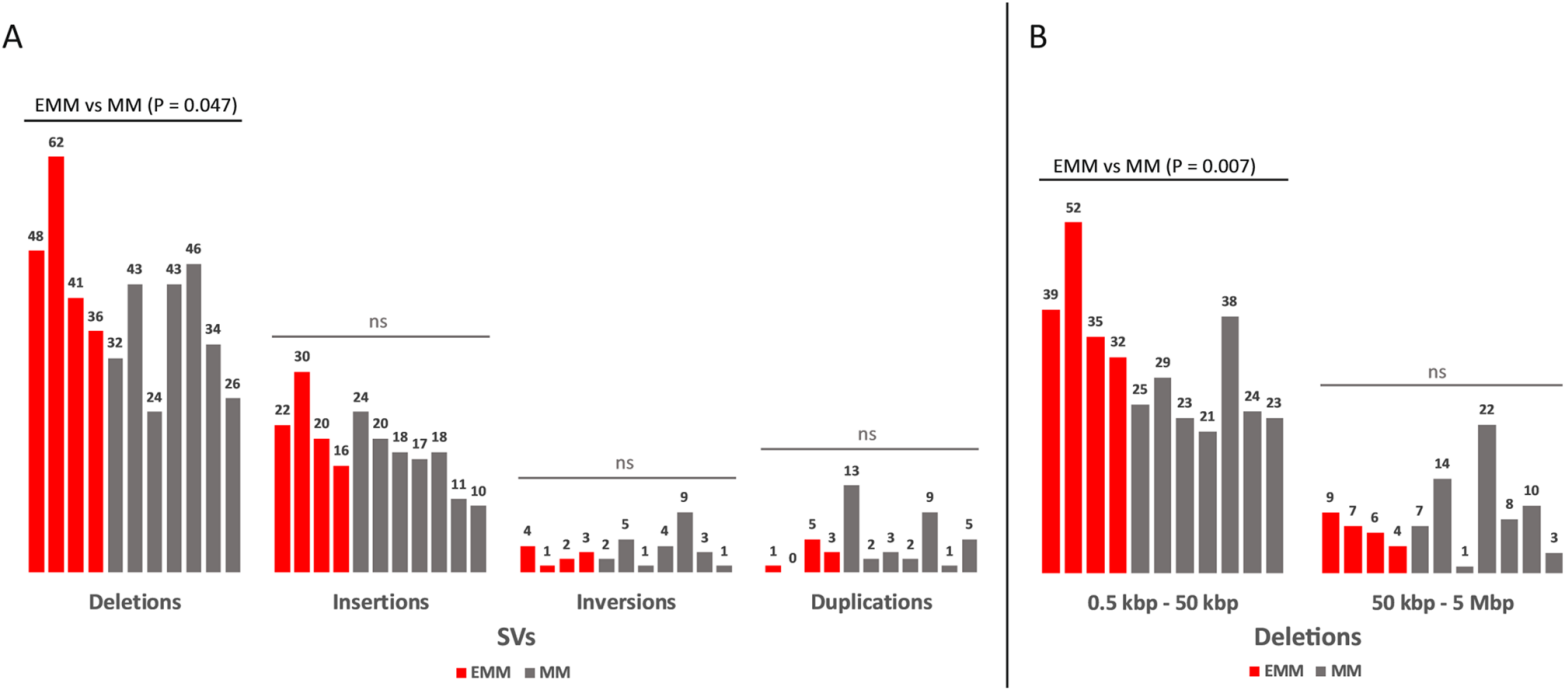
(**A**) Distribution of SVs (deletions, insertions, inversions and duplications) and (**B**) deletions subdivided according to their size in EMM (red columns) and MM (grey columns) patients. Each column represents an individual patient and the column height the number of SVs detected.

The median number of SVs per patient was as follows: deletions, 1700 (min–max: 1583–1755); insertions, 4433 (4268–4550); inversions, 62 (44–75); duplications, 54 (48–79); chromosome translocations, 2 (0–8); and intrachromosomal rearrangements, 6 (0–24) (Table S5). After filtering only for likely somatic variants, the number of deletions per patient (41, 24–62) dominated over insertions (18, 10–30), inversions (3, 1–9) and duplications (3, 0–13) (Table S5, Fig. 2A), reaching high inter-individual variability. All detected chromosome translocations and intrachromosomal rearrangements were identified as somatic-like in all samples.

The EMM genome contained more deletions than the MM (median number of 45 vs 34, P = 0.05), particularly small deletions of 500 bp–50 kbp (37 vs 24, P = 0.01) (Fig. 2). The number of inversions and duplications did not differ between EMM and MM (P > 0.05). The spectrum of SVs and affected genes and chromosomes displayed high inter-individual variability. In addition to the deletion of the *CCSER1* gene on chromosome 4 found in ~ 45% of our patients, the SVs in two patients covered *NKAIN2*, and two others covered the *EYS* gene, both within a commonly affected region, 6q.

Regarding CNVs, losses in copy numbers (CN = 1) (median per patient 13, min–max 5–38), as well as gains (CN = 3–25) (37, 4–56), were common in all patients. Except for two MM patients, the majority of patients had a mean of five regions of CN > 3 (range 1–16 per patient) in their genomes. The distribution of CNVs across the genome was highly variable in enrolled EMM and MM patients.

Optical mapping confirmed 98% of SV and CNV changes detected by diagnostic cytogenetic and arrayCGH assessments (Tables S2, Tables S3) and revealed numerous novel rearrangements in all enrolled patients.

### Interchromosomal translocations in MM and EMM

In three MM patients, optical mapping detected translocations within *IGH/IGK/IGL* immunoglobulin loci, t(4;14) and t(11;14) (confirmed by diagnostic FISH), and one t(8;22)(q24;q11) translocation that was detected by mapping only (this region is not routinely assessed by FISH). In EMM patients, no translocations within *IGH/IGK/IGL* immunoglobulin loci were detected.

**Figure 3:**
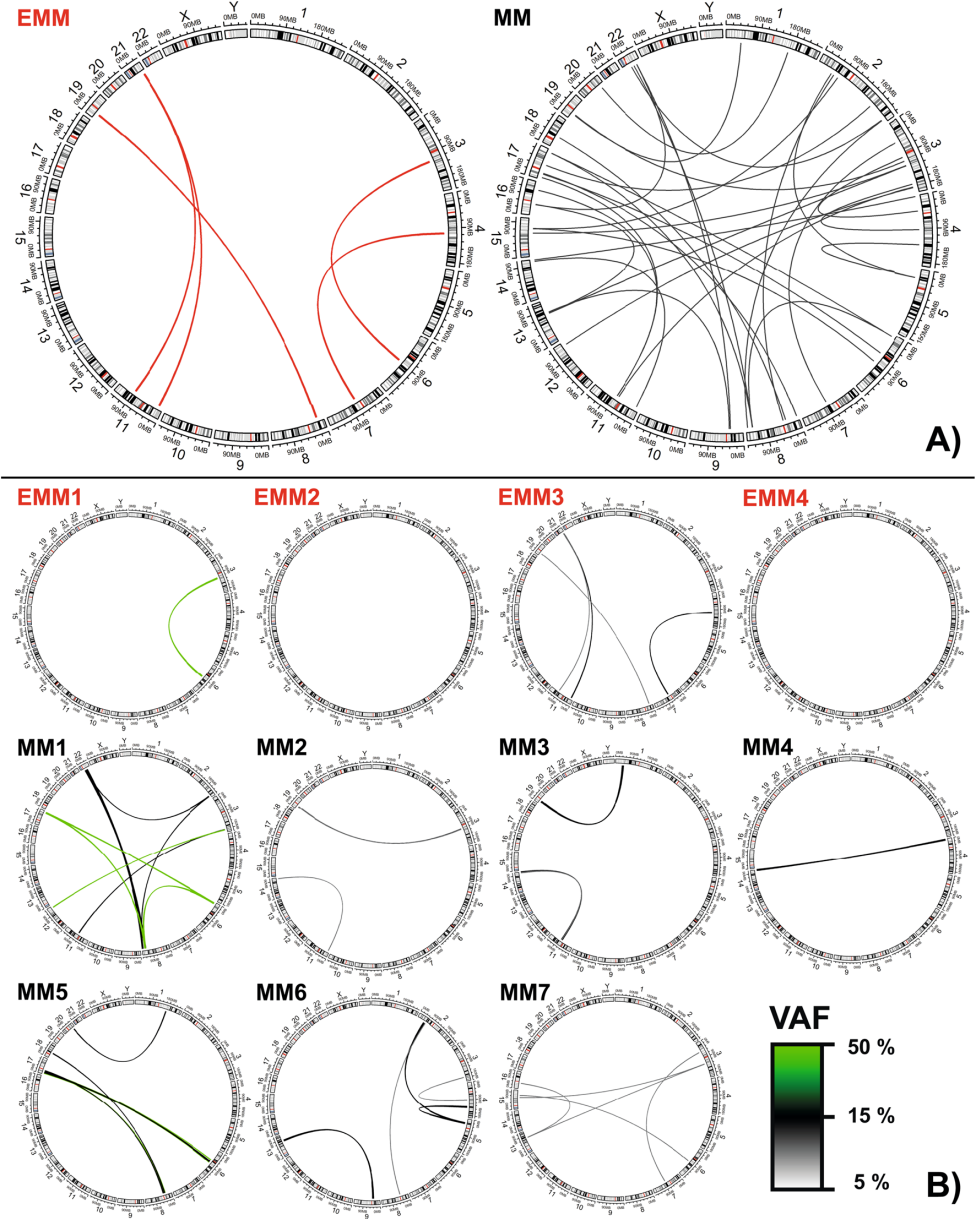
Distribution of chromosome translocations in EMM (red lines) and MM (black lines) patients. Large circos plots (**A**) show the sum of translocation in EMM and MM groups; (**B**) small circos show detected translocations in a particular patient. The VAF of each translocation is denoted by the thickness and colour of the line (key bottom right). SVs were visualised using circos plots^28^.

Additionally, numerous other translocations were detected across all MM patients, frequently affecting chromosomes 2, 3, 6 and 8 (Table S6). All MM patients carried at least two translocations, except for one MM patient with only t(4;14) (Table S6, Fig. 3). Complex chromosomal rearrangements involving three chromosomes were detected in four (57%) MM patients but not in any EMM patients (Table S6, Fig. 3). The translocations were present at clonal and subclonal levels (VAF 5–43%). The affected genes and putative fusion genes are shown in Table S6.

EMM genomes were associated with fewer translocations than MM; two EMM patients had no translocations, one EMM patient had one translocation and the only EMM patient that reached complete response after first-line therapy had four translocations. The translocations were present at clonal and subclonal levels (VAF 5–49%) (Fig. 3).

### Intrachromosomal rearrangements in MM and EMM

**Figure 4:**
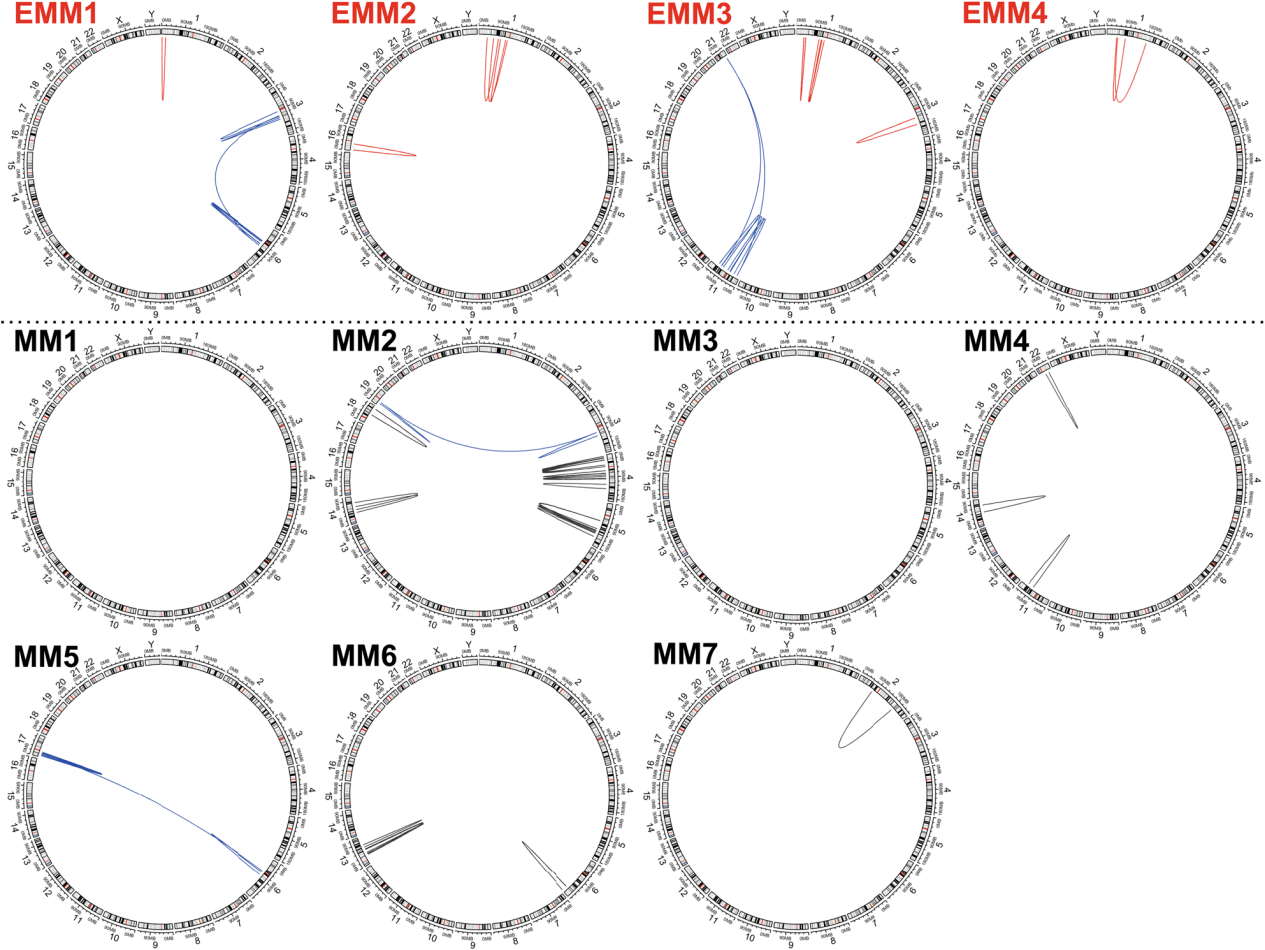
Intrachromosomal rearrangements identified in EMM (red lines) and MM (black lines) patients. Blue lines represent complex rearrangements including translocations.

Large chromosomal rearrangements encompassing regions longer than 5 Mbp on chromosome 1 were detected in all EMM genomes but not in any MM genomes (Fig. 4, Table 2). The large rearrangements, together with the small SVs (predominantly deletions), affected various regions across chromosome 1, often involving deletions and inversions accompanying the CNV changes. EMM1 had one large intrachromosomal rearrangement of 14.5 Mbp, encompassing 230 genes in the 1p36 region, and five deletions; EMM2 had three large intrachromosomal rearrangements of 47.5 Mbp, 57.9 Mbp and 21.5 Mbp, encompassing 1093 genes in the 1p35-p31, 1p32-p12 and 1p22-p13 regions, and an additional six deletions and one insertion. EMM3 had four rearrangements on chromosome 1 of 7.6 Mbp, 7.5 Mbp, 12.6 Mbp and 12.8 Mbp, encompassing 794 genes in the 1p35-p34, 1p22-p21 and 1p21-p13 regions, and two deletions. EMM4 had two large rearrangements of 36.1 Mbp and 12.0 Mbp, encompassing 564 genes in the 1p34-p31 and 1p34-1q23 regions, three deletions and five insertions (Fig. S1). The majority of the affected genes by intrachromosomal rearrangement across chromosome 1 in EMM were associated with cancer (~ 35%), cell cycle (~ 30%) and inflammation (~ 10%); very few affected genes were associated with MM (~ 10%) (Table S7).

**Table 2:** SVs on chromosome 1 in enrolled EMM and MM patients.

ID	SV type	SV size (kbp)	Cytobands	Chromosome A		Chromosome B		VAF (%)	Number of affected genes
				Nr	RefStart	Nr	RefEnd		
EMM1	Intra-chrom	14,502	1p36.33-p36.13	1	1451742	1	16010418	8	230
	Deletion	12.3	1p32.3	1	54437326	1	54468426	13	0
	Deletion	0.7	1p31.1	1	73986848	1	73989906	49	0
	Deletion	0.7	1q21.2	1	149255041	1	149297922	12	0
	Deletion	83.9	1q31.1	1	188862967	1	188948998	13	0
	Deletion	0.9	1q41	1	219227885	1	219230864	69	0
EMM2	Intra-chrom	46,876	1p35.1-p31.1	1	33649122	1	80528558	13	475
	Deletion	1,611	1p32.3	1	52146402	1	53764220	6	22
	Deletion	20.0	1p32.2	1	56859220	1	56897293	5	1
	Deletion	311	1p32.2	1	57042204	1	57356850	6	1
	Insertion	0.6	1p32.2	1	57981404	1	57987615	27	1
	Intra-chrom	57,947	1p32.1-p12	1	60494258	1	118466419	9	443
	Deletion	14.5	1p31.1	1	70404062	1	70438713	20	1
	Deletion	5.5	1p31.1	1	70766057	1	70784216	25	0
	Intra-chrom	21,501	1p22.2-p13.3	1	89446367	1	110953619	6	175
	Deletion	7.3	1q25.3	1	182529994	1	182549190	61	1
EMM3	Intra-chrom	7,575	1p35.2-p34.3	1	30221701	1	37801246	15	120
	Intra-chrom	7,465	1p35.2-p34.3	1	30860870	1	38353947	8	126
	Intra-chrom	12,639	1p22.1-p21.1	1	93051297	1	105699124	9	81
	Deletion	1.3	1p22.1	1	93329493	1	93335818	10	1
	Intra-chrom	12,839	1p21.1-p13.2	1	102272272	1	115119846	21	140
	Deletion	5.8	1q42.3	1	235342739	1	235355292	18	1
EMM4	Deletion	53.1	1p36.33	1	1679533	1	1743791	7	3
	Insertion	2.5	1p36.12	1	21983384	1	22006562	13	2
	Intra-chrom	36,076	1p34.2-p31.1	1	39891936	1	75973164	7	363
	Deletion	3,687	1p34.2-p34.1	1	42633541	1	46350766	8	102
	Intra-chrom	119,844	1p34.1-1q23.3	1	44054030	1	163946030	8	1175
	Insertion	2.5	1p13.2	1	111794239	1	111807945	11	1
	Insertion	6.1	1p13.1	1	115530277	1	115543191	14	0
	Deletion	0.8	1q31.3	1	195460336	1	195473816	16	0
	Insertion	18.6	1q32.2	1	207515921	1	207534396	12	2
	Insertion	6.0	1q32.3	1	213171761	1	213205644	12	1
MM1	Insertion	5.9	1p36.33	1	1590522	1	1654114	17	7
	Insertion	59.9	1q21.2	1	149365317	1	149390055	51	0
	Insertion	10.9	1q21.3	1	152289954	1	152296885	13	0
	Deletion	231.9	1q25.1	1	175947709	1	176185749	28	2
	Deletion	3176	1q32.3-q41	1	214386812	1	217572960	21	8
	Insertion	19.9	1q42.12	1	226337005	1	226338164	32	0
	Deletion	315.8	1q43	1	238186340	1	238513554	23	1
	Deletion	31.4	1q43	1	239651938	1	239694630	23	1
MM2	Insertion	5.4	1p36.12	1	20372589	1	20396493	12	1
	Deletion	14.6	1p31.1	1	83171695	1	83186312	19	0
	Duplication	95.5	1q21.2	1	148669395	1	148764931	46	1
	Insertion	2.4	1q23.2	1	161184787	1	161193876	13	1
	Deletion	4.4	1q25.2	1	179360227	1	179368480	28	1
MM3	t(1;19)		1p34.3-19p13.11	1	38291095	19	16838518	15	
	t(1;19)		1p34.3-19p13.12	1	39900616	19	15299192	18	
	Deletion	0.6	1q32.1	1	200212204	1	200225458	24	0
MM4	Insertion	14.6	1p36.31	1	5999446	1	6006996	20	0
	Insertion	1.8	1p12	1	119153160	1	119157652	27	0
	Duplication	95.5	1q21.2	1	148669395	1	148764931	49	1
	Insertion	2.4	1q23.3	1	161184787	1	161193876	28	1
MM5	Insertion	23.0	1p36.13	1	16040685	1	16054506	9	0
	Deletion	1.8	1p34.2	1	39074813	1	39085202	18	1
	Deletion	5.4	1p31.3	1	62279900	1	62311886	20	1
	Deletion	0.6	1p31.1	1	72996895	1	73015474	24	0
	Duplication	79.6	1q24.2	1	168300624	1	168380191	60	2
	Insertion	199.5	1q24.2	1	168300624	1	168380191	34	2
	t(1;20)		1q24.3-20q13.2	1	170346977	20	53385582	12	
	Duplication	57.9	1q42.13	1	227147621	1	227205509	19	1
MM6	Insertion	3.2	1p34.3	1	37899628	1	37903954	15	1
	Deletion	14.6	1p31.1	1	83171695	1	83190595	17	0
MM7	Insertion	5.7	1p36.33	1	1590522	1	1654114	5	0
	Insertion	3.8	1p36.32	1	4070359	1	4096038	5	0
	Deletion	23.2	1p36.12	1	21983384	1	22006562	17	2
	Insertion	3.4	1p34.1	1	44413885	1	44419138	7	1
	Duplication	47.2	1p21.3	1	99166139	1	99213371	7	0
	Insertion	55.4	1p21.3	1	99180335	1	99199495	6	0
	Deletion	721.6	1q21.1	1	143310164	1	144170341	25	9
	Duplication	633.7	1q22-q23.1	1	156251622	1	156885360	5	28
	Deletion	3.0	1q24.1	1	166768561	1	166777221	16	0
	Deletion	0.7	1q32.1	1	200212204	1	200225458	15	0

*intra-chrom* intrachromosomal rearrangements, *VAF* variant allele frequency, *SVs* structural variants.

In contrast, no intrachromosomal rearrangements, fewer deletions (2, 0–4) and more insertions and duplications (4, 0–6) on chromosome 1 were detected in MM compared with EMM. The number of affected genes was also low (2, 0–40).

Additionally, intrachromosomal rearrangements were distributed across other chromosomes in both MM and EMM (Table S8, Fig. S2). The typical patterns of intrachromosomal translocation were large deletions with partial inversion, accompanied by copy number loss. Multiple rearrangements within the same chromosome often occurred in some patients. In four patients, these rearrangements were part of the interchromosomal translocations (highlighted in blue in Fig. 4).

### SVs and CNVs in high-risk loci associated with MM/EMM

In addition, we focused on SVs in high-risk regions such as *IGH/IGK/IGL* immunoglobulin loci, del(17p13), del(13q14), the 8q24 region, 1q21 gain and del(1p32).

Regarding the *IGH* locus, optical mapping revealed t(4;14) and t(11;14) in three MM, which were confirmed by cytogenetics. In the majority (6/7) of MM samples, but not in any EMM sample, translocations involving immunoglobulin-associated chromosomes 2, 14 and 22 were detected. Additionally, a 0.4 Mbp inversion was detected in one EMM patient, and 1.2 Mbp and 0.8 Mbp duplications on chromosome 14 in two MM patients (Table S9). Also, somatic-like SVs within the *IGK* and *IGL* loci were detected: deletions in five patients (three EMM and two MM), insertions in two (two EMM) and duplication in one (MM) were identified (Table S9, Fig. S3).

**Figure 5:**
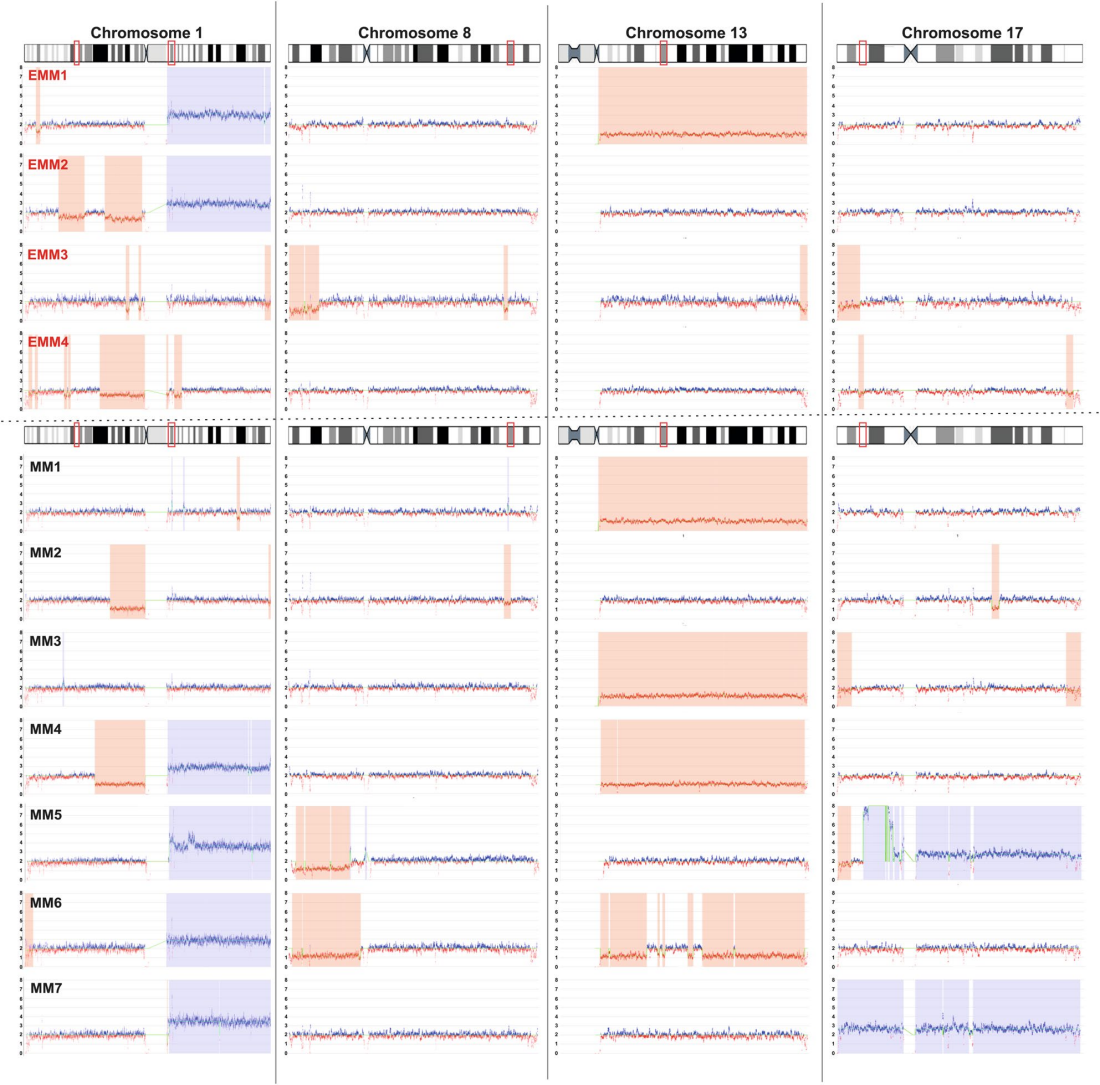
The genome CNVs on chromosomes 1, 8, 13 and 17 in EMM (upper part) and MM (lower part) patients. Blue indicates gains and red indicates losses in gene copy numbers. The vertical bars represent detected copy number aberrations. The red boxes on the ideogram highlight the high-risk regions.

Regarding *TP53* disruption, diagnostic analysis by FISH and NGS did not detect any abnormalities in enrolled patients. Nevertheless, optical mapping revealed copy number loss (CN = 1) in the region overlapping the *TP53* gene in two EMM patients (Fig. 5).

Optical mapping confirmed del(13q14) identified by FISH in one EMM and three MM patients. Additionally, optical mapping detected a 1.1 Mbp deletion affecting the *RB1* gene, supported by copy number loss in the 13q14.2 region in one MM patient, which was not detected by FISH (Fig. 5).

Regarding the 8q24 locus, one EMM patient carried a deletion and one MM a duplication, detected by both mapping and cytogenetics. Optical mapping revealed additional changes within this locus associated with *MYC* gene amplification in three patients: one MM patient had a 0.6 Mbp insertion and three translocations, t(6;8), t(8;17) and t(8;22); one MM had an inversion; and one EMM patient had a novel 0.2 Mbp insertion (Table S10).

Regarding high-risk regions on chromosome 1 commonly affected in MM, we confirmed 1q21 gain in six patients (two EMM and four MM) and del(1p32) in one EMM patient (Table S11). On chromosome 1, 1.4 times more SVs within/outside the high-risk 1q21/1p32 regions were found in EMM than in MM. In EMM, deletions (50%) and intrachromosomal rearrangements (31%) were the most frequent, not duplications and translocations.

## Discussion

This study characterised genomes of BM myeloma cells in newly diagnosed EMM and MM patients using next-generation optical mapping. When comparing the EMM and MM genomes, EMM was associated with large intrachromosomal rearrangements across chromosome 1, fewer interchromosomal translocations and more deletions across the entire genome compared with MM. For high-risk loci, optical mapping revealed copy number loss in the 17p13 region in two EMMs, numerous SVs and CNVs in other high-risk 8q24 and 13q14 regions and *IGH/IGK/IGL* immunoglobulin loci that were not detected by diagnostic cytogenetic evaluation.

To date, the complex genomic architecture in MM and EMM has been poorly characterised, probably due to its complexity, heterogeneity and multiple levels of somatic mosaicism^29,30^. Therefore, we analysed EMM and MM genomes using innovative optical mapping that can detect small SVs and CNVs as well as complex large genomic rearrangements or chained fusions^14,31–33^, which are not recognisable by NGS and/or cytogenetics. The utility of this approach has been recently shown in leukaemia samples, where optical mapping confirmed the results of whole-genome sequencing and/or cytogenetic analysis and additionally revealed a large number of SVs not previously recognisable in analysed samples^14,33^. In this study, we used optical mapping for the first time to study the genome architecture of isolated myeloma cells from BM from newly diagnosed EMM and MM patients.

In line with the high degree of somatic genomic mosaicism and multiple levels of genetic variation in MM^29,30^, long-fragment mapping revealed simple and complex genomic rearrangements and CNVs in all samples. More interchromosomal translocations were detected in MM patients than EMM. Except for one patient with a high-risk 14q32 translocation, a common primary event in MM^34^, all MM patients had at least two other translocations. These often involved chromosomes 2, 3, 6 and 8, and many of them led to gene disruptions or the creation of putative gene fusions with at least one partner associated with cancer. Moreover, interchromosomal translocations in MM were often accompanied by intrachromosomal rearrangements located in the same chromosomal loci. On the contrary, three EMM patients had one or zero translocations, and one EMM patient with good treatment response had three translocations; the translocations occurred in our patients at subclonal and clonal levels (5–49%). Although the impact of clonal status on the prognostic value of SVs is unclear for most cancers, recent NGS studies in MM have shown that the clonality status of mutations does not influence survival but does impact the disease phenotype^35^. Experimental evidence also suggests that MM progression, both spontaneous in asymptomatic stages and at relapse after treatment, is linked to its heterogeneous subclonal composition^36^; thus, the direct measures of the clone size and its intrinsic biological features deserve future investigation. Optical mapping also revealed numerous complex translocations, involving three chromosomes in about half of the MM patients but not in the EMM patients. There are already reports about large chromosomal rearrangements, called chromothripsis, in MM^29,30^. Such complex structural changes, often accompanied by loss of heterozygosity^37^, are difficult to identify by other techniques and may escape attention. The presence of unusual rearrangements of numerous chromosomes in MM, but not EMM, deserves future investigation.

In addition to interchromosomal translocations, we detected numerous intrachromosomal rearrangements, which are rearrangements that involve loci located on the same chromosome. To date, few cancer types harbour both interchromosomal and intrachromosomal rearrangements; one of them is MM^38^. It has been suggested that the occurrence of intra- or interchromosomal recombinations depends on the spatial proximity between recombinogenic partners within the chromosome territories (CTs), a non-randomly formed, distinct space where each chromosome decondenses^39,40^. When loci are situated near the surface of their CTs, interchromosomal translocations occur, and when they are located deep in the CTs, intrachromosomal rearrangements occur^41^. There is already evidence that chromosomes involved in commonly occurring translocations – t(4;14), t(14;16) and t(11;14) – in MM are located within overlapping CTs^42,43^; however, the mechanisms of intrachromosomal rearrangements have not been investigated in MM.

Importantly, we detected EMM-specific intrachromosomal rearrangements encompassing several Mbp-long regions within chromosome 1, commonly including combinations of deletions and inversions and affecting hundreds of genes. These rearrangements were located across the whole of chromosome 1 and led to changes in the copy number of genes on large regions of this chromosome. The intrachromosomal rearrangements on chromosome 1 have already been reported in progressive, multi-drug refractory EMM^10^ and EMM with soft tissue involvement at the time of MM diagnosis^44^. Interestingly, 80% (8/10) of patients with soft tissue EMM had chromosome 1 abnormalities, and an association between chromosome 1 abnormalities and soft tissue EMM was suggested^44^. Furthermore, 1p deletion and/or 1q gain were associated with the extramedullary plasmablastic transformation of MM in both BM and matched extramedullary tissue^45^. Other studies reported an association of chromosome 1 abnormalities in MM with the relapsed disease^46^. The affected patients have an exceedingly poor prognosis, short progression-free survival and overall survival, even in the era of novel therapies^44,47,48^. A recent study showed that the adverse impact of chromosome 1 abnormalities on survival is of similar magnitude to other high-risk chromosomal abnormalities^47^. The crucial role of chromosome 1 in MM pathogenesis is also supported by the significant overrepresentation of genes derived from chromosome 1 in the high-risk signature in MM^48^. The occurrence of fewer interchromosomal translocations and more intrachromosomal rearrangements in EMM, particularly on chromosome 1, suggests that recombinations within loci deep in CTs may play a crucial role in MM pathogenesis, particularly influencing the phenotype of the disease. Furthermore, the observed chromosome 1 abnormalities may play a role on the required events that allow the dissemination of myeloma cells from BM to blood and distant tissues; this also deserves future investigation.

In addition to translocations and intrachromosomal rearrangements, we also detected tens of SVs in every EMM and MM genome. The most common were deletions distributed across all chromosomes. In particular, deletions ranging in size from 500 bp to 50 kbp occurred more frequently in EMM than in MM. An increased number of deletions in MM has already been associated with MM progression, as shown by comparing MM genomes at diagnosis and relapse^49^. Future studies should investigate the relationship of a higher deletion load in EMM compared with MM as well as prognosis. We also detected numerous novel SVs and CNVs within high-risk loci associated with MM not previously detected by sequencing and cytogenetics.

The most critical genetic factors that portend a poor prognosis for MM are translocations within the *IGH/IGK/IGL* loci^50^. Our study confirmed the common translocations t(4;14) and t(11;14) in three MM patients and revealed additional interchromosomal translocations involving chromosomes 2, 14 and 22, where immunoglobulin genes are located, in a majority (6/7) of MM patients. The functional consequences of the translocations outside the *IGH/IGK/IGL* loci needs to be clarified, as they may influence antibody expression and function and the mediation of disease phenotypes. Interestingly, we did not detect any translocation on the previously mentioned chromosomes in EMM, where deletions and inversions were predominantly found. Differences between MM and EMM in genetic rearrangements on immunoglobulin-associated chromosomes should be further investigated.

Next, we were interested in the disruption of the 17p13 locus overlapping the *TP53* gene, a driver aberration associated with EMM^51,52^, poor prognosis and low treatment response rates in MM patients^53^. The loss of *TP53* and other genetic aberrations may additionally occur in the extramedullary mass due to regional clonal evolution, as shown by comparing extramedullary tumours with their BM myeloma cells^7,13^. Although no *TP53* disruption was detected in myeloma cells from BM aspirates of enrolled patients by diagnostic cytogenetic and mutational analyses, optical mapping revealed copy number loss in the 17p13 region in two EMM patients. Our data further support the key role of *TP53* in EMM and emphasise the need to routinely incorporate SVs and CNVs, the major forms of genetic alterations in cancer, at many length scales to understand the MM genome more comprehensively.

Optical mapping also confirmed rearrangements at the *MYC*/8q24 locus, a late tumour progression event associated with an increased expression of *MYC* and poor prognosis^54^, in about a third of patients. One MM patient had three translocations within this region, and two others had SVs within the *MYC*/8q24 locus. Whether the changes at the *MYC*/8q24 locus were EMM specific, as reported by others^55^, needs further investigation using larger cohorts.

This study has several limitations. First, we did not investigate extramedullary tumour mass because invasive biopsy was not feasible in enrolled patients. Second, due to the moderate number of patients included in this exploratory study, a sub-analysis based on clinical and laboratory parameters was not performed. Third, the proportion of the IgA subtype was higher in the MM cohort. However, there is growing evidence that adverse prognosis in patients with IgA MM versus non-IgA MM subtypes is more likely to be caused by the misclassification of disease response or the delayed detection of disease due to an underestimation of tumour burden^56^ than changes in expression profile or cytogenetics^57–59^. Future studies on larger patient cohorts enabling a subanalysis of patients with particular clinical characteristics and stages of disease and the investigation of extramedullary tissue sites are warranted.

There is a growing body of evidence on the utility of optical mapping for comprehensive SV detection in haematology and solid tumours^14,31–33^. The aberrations detected by mapping have been confirmed by cytogenetics^14^ or NGS^33,60^, particularly by long-read sequencing^61,62^, as also shown in our study. Optical mapping thus provides an ideal complement to sequencing for resolving complex genomic architecture in cancers^31^.

## Conclusion

Our pilot study using next-generation optical mapping revealed that in addition to known high-risk cytogenetic factors, chromosome 1 abnormalities in BM myeloma cells are associated with extramedullary progression. The detection of numerous novel, distinct genetic aberrations associated with EMM and MM shows the potential of optical mapping for the refinement of complex genomic architecture in MM and its phenotypes. The methodology and results described here represent a significant advance that may accelerate the introduction of genomics at long-length scales into clinical decisions for MM.

## Supplementary Information

Supplementary Information.

## Data Availability

The data of this study are available from the corresponding author on reasonable request.

## Abbreviations

EMM: Extramedullary multiple myeloma
MM: Multiple myeloma
BM: Bone marrow
HMW DNA: High molecular weight DNA
FISH: Fluorescence in situ hybridization
NGS: Next-generation sequencing
BMMC: Bone marrow mononuclear cell
SV: Structural variant
VAF: Variant allele frequency
CNV: Copy number variation
FICTION: Fluorescence immunophenotyping and interphase cytogenetics as a tool for investigation of neoplasms
CT: Chromosome territory
