# Supplementary material for: Whole-genome optical mapping of bone-marrow myeloma cells reveals association of extramedullary multiple myeloma with chromosome 1 abnormalities
Source: Sci Rep. 2021 Jul 19;11:14671. doi: 10.1038/s41598-021-93835-z (PMC8289962; doi:10.1038/s41598-021-93835-z)

Supplementary file

Whole-genome optical mapping of bone-marrow myeloma cells reveals association of extramedullary multiple myeloma with chromosome 1 abnormalities

Eva Kriegova, Regina Fillerova, Jiri Minarik, Jakub Savara, Jirina Manakova, Anna Petrackova, Martin Dihel, Jana Balcarkova, Petra Krhovska, Tomas Pika, Petr Gajdos, Marek Behalek, Michal Vasinek, Tomas Papajik

Supplementary content:

Table S1 Clinical characteristics of enrolled EMM and MM patients.

Table S2 Characteristics of MM and EMM patients detected by cytogenetics (karyotyping, FISH) and gene mutations (NGS).

Table S3 Copy number variations identified by arrayCGH.

Table S4 Run metrics of optical mapping and quality control parameters of analysed samples.

Table S5 Numbers of structural variants (SVs) and likely somatic SVs detected by optical mapping.

Table S6 Interchromosomal translocations detected in EMM and MM by optical mapping.

Table S7 SVs on chromosome 1 in enrolled MM and EMM patients.

Table S8 Intrachromosomal rearrangements across all chromosomes in MM and EMM samples.

Table S9 SVs within immunoglobulin *IGH/IGK/IGL* loci in MM and EMM patients.

Table S10 SVs and CNVs within *8q24/MYC* gene region detected by optical mapping, FISH and arrayCGH.

Table S11 CNVs on chromosome 1 in MM and EMM samples detected by optical mapping.

Fig. S1 Intrachromosomal rearrangements (> 5Mb) within chromosome 1 in EMM patients.

Fig. S2 Intrachromosomal rearrangements on chromosomes 4 and 14 – representative example.

Fig. S3 Genomic aberrations within *IGH/IGK/IGL* immunoglobulin loci in EMM patients – representative examples.

Table S1 Clinical characteristics of enrolled EMM and MM patients.

Patient ID	Gender	ISS	Durie-Salmon stage	LC + FLC	Type of treatment	Response to treatment	Other events	Cell counts in BM 10^6 bb/ml	CD138+ cell infiltration in whole BM (%)*	CD138+ cell infiltration after enrichment* (%)
**EMM1**	Male	3	IIIA	IgG kappa	IsaVRD	VGPR	No	16	30	90
**EMM2**	Male	3	IIIB	IgG kappa	VMP+CTD	VGPR	No	20	5	81
**EMM3**	Female	1	IIA	IgA lambda	IsaVRD	CR	No	14	27	98
**EMM4**	Male	1	IIIA	IgG kappa	VTD+ASCT	PR	No	12	3	84
**MM1**	Female	1	IIA	IgG kappa	MP	PR	MDS	114	16	90
**MM2**	Male	2	IIIA	IgA kappa	VRD	CR	MDS	17	10	82
**MM3**	Male	3	IB	IgG kappa	VRD	CR	No	30	5	87
**MM4**	Male	1	IIA	IgA kappa	VTD+ASCT	VGPR	No	7	29	86
**MM5**	Male	3	IIIA	IgA kappa	VMP	VGPR	No	3	20	83
**MM6**	Male	3	IIA	IgA kappa	VMP	CR	No	3	8	82
**MM7**	Female	1	IA	IgA lambda	RD	PR	No	74	36	96

ISS: International Staging System, **LC**: monoclonal protein's light chain, **FLC**: free light chain, **VRD**: bortezomib-lenalidomide-dexamethasone, **IsaVRD**: isatuximab-bortezomib-lenalidomide-dexamethasone, **VMP**: bortezomib-melphalan-prednisone, **CTD**: cyclophosphamide-thalidomide-dexamethasone, **VTD**: bortezomib-thalidomide-dexamethasone, **ASCT**: autologous stem cell transplantation, **RD**: lenalidomide-dexamethasone, **MP**: melphalan-prednisone, **VGPR**: very good partial response, **PR**: partial response, **CR**: complete response, **MDS:** myelodysplastic syndrome, **BM:** Bone-marrow. *infiltration CD138+ cells is given based on the immunophenotyping: all enrolled patients had more than 10% infiltration of plasma cells found in BM smears.

Table S2 Characteristics of MM and EMM patients detected by cytogenetics (karyotyping, FISH) and gene mutations (NGS).

Patient ID	Cytogenetics								Pathogenic mutation (NGS)			
	IgH transl	del(17p)	del(13q14)	del(1p32)	gain(1q21)	Monosomy	Trisomy	Tetrasomy	*TP53*	*KRAS*	*NRAS*	*BRAF*
**EMM1**	No	No	Yes	No	Yes	13	4,11,18,19,21	15	No	Yes	No	No
**EMM2**	No	No	No	Yes	Yes	No	3,5,9,11,19,21	No	No	No	No	No
**EMM3**	No	No	No	No	No	X	3,9,11	15	No	No	No	Yes
**EMM4**	No	No	No	No	No	No	5,11,15,19,21	9	No	No	No	No
**MM1**	No	No	Yes	No	No	13	5,7,9,15,19	No	No	No	No	Yes
**MM2**	No	No	No	No	No	No	3,9,11,15,18,19	21	No	No	No	No
**MM3**	t(11;14)	No	Yes	No	No	13,22	No	No	No	No	No	No
**MM4**	t(4;14)	No	Yes	No	Yes	13,Y	No	No	No	No	No	No
**MM5**	No	No	No	No	Yes	No	2,3,5,9,11	15	No	No	No	No
**MM6**	No	No	No	No	Yes	No	9,11,15,19,21	No	No	Yes	No	Yes
**MM7**	No	No	No	No	Yes	No	5,11,15,17,18,19	No	No	No	No	No

No: no genetic aberration found.

Table S3 Copy number variations identified by arrayCGH.

Patient ID	Chromosome 1		Chromosome 17		Other chromosomes	
	Loss	Gain	Loss	Gain	Loss	Gain
**EMM1**	No	1q21.1-1q44	No	No	6q13-6q14.1, 6q15-6q27, 20q12	3p26.3-3p11.1, 3q13.2-3q13.31, 3q13.33, 3q21.3, 3q22.1, 5p15.33-5p11, 6p25.3-6p11.1, 6q11.1-6q12, 6q14.1, 6q14.3-6q15
**EMM2**	1p35.1-1p32.1, 1p31.1-1p12	1q21.1-1q44	No	No	16q11.2-16q24.3	9p24.3-9p11.2
**EMM3**	1p35.2. 1p34.3, 1p21.1, 1p13.2-1p12	No	17q21.3	No	2p13.3-2p13.2, 6q13, 8p23.3-8p22, 8q24.13-8q24.21, 19q13.12-19q13.13	19p13.3-19p11, 21q11.2-21q21.1, 21q22.12-22q22.3
**EMM4**	1p34.2-1p33, 1p31.1-1p11.2, 1q21.1, 1q21.3-1q23.1	No	No	No	16p13.3	No
**MM1**	No	No	No	No	16q11.2-16q24.3, 18p11.32	No
**MM2**	1p22.3-1p11.2	No	No	No	2q36.1, 2q36.1-2q36.3, 4p16.3-4p16.2, 4p15.31, 4q13.3-4q21.1, 4q21.22-4q22.1, 4q22.3-4q23, 4q26-4q27, 4q31.1-4q31.2, 8q24.13-8q24.21	No
**MM3**	No	No	No	No	No	No

**MM4**	1p31.1-1p12	1q21.1-1q44	No	No	2q11.1-2q37.3, 6q25.2-6q27, 7p22.3-7p11.2, 11q14.1-11q22.3, 14q22.1-14q31.1, 20p13-20p11.1, 22q11.23-22q13.1, 22q13.12-22q13.33	Xq27.3-Xq28
**MM5**	No	1q21.1-1q44	No	17p13.1, 17p13.1-17p12, 17p11.2-17p11, 17q11.1-17q25.13	8p23.3-8p12	6p25.3-6p11.1, 6q11.1-6q12, 6q12, 6q13-6q14.1, 6q14.1, 18q12.2-18q12.3, 18q22.1-18q22.3, 19p13.3p1
**MM6**	No	1q21.1-1q44	No	No	6q22.31-6q27, 8p21.2-8p12, 13q12.13-13q13.3, 13q21.2, 13q22.1-13q31.1, 13q32.1-13q33.2	5p15.33-5p13.3, 9q13-9q34, Xq21.31-Xq28
**MM7**	No	1q21.1-1q44	No	No	4p16.3-4p13, 6q15-6q27, 9q34.3, Xp22.33-Xp11.1, Xq11.1-Xq27.3	3p26.3-3p21.1,3q11.2-3q26.2, 3q26.31-3q29, 6p25.3-6p12.2, 9p24.3p11.2, 9q12-9q34.3, 9q34.3, 10p14-10p13, 20q11.22, 22q13.1, Xq27.3-Xq28

No: no genetic aberration found.

Table S4 Run metrics of optical mapping and quality control parameters of analysed samples.

Patient ID	Throughput (>20kbp)	Throughput (>150kbp)	Map rate	Effective coverage	Label density /100kbp	N50 (>20 kbp)	N50 (>150 kbp)	PLV (3-10 %)	NLV (6-15 %)
**EMM1**	539	482	89	134	17	0.409	0.446	4	7
**EMM2**	630	480	93	142	17	0.258	0.322	9	8
**EMM3**	702	481	51	78	23	0.269	0.421	3	10
**EMM4**	2406	1257	80	324	14	0.160	0.219	3	15
**MM1**	521	427	90	120	16	0.320	0.378	9	8
**MM2**	726	481	89	134	15	0.191	0.244	3	8
**MM3**	2235	611	64	127	18	0.091	0.243	3	6
**MM4**	1465	794	63	162	19	0.174	0.339	3	10
**MM5**	702	481	89	134	15	0.200	0.253	3	13
**MM6**	908	481	70	106	18	0.160	0.285	4	10
**MM7**	2629	1710	42	230	18	0.231	0.326	3	12

Throughput: The amount of DNA that is detected per sample during the run. **Map rate:** The percentage of molecules that map to the reference for molecules ≥ 150 kbp. **Effective coverage:** Average Map Rate x Total DNA / length of the reference. **Label density**: The number of labels that are detected by the image detection algorithm per 100 kbp of DNA length for molecules ≥ 150 kbp. **N50:** The molecule length N50 for all molecules that are ≥ 150 kbp/20 kbp in length. **PLV** (Positive Label Variance): Percentage of molecule labels absent in reference labels. **NLV** (Negative Label Variance)**:** Percentage of reference labels absent in molecule labels.

Table S5 Numbers of structural variants (SVs) and likely somatic SVs detected by optical mapping.

Patient ID	Insertions		Deletions		Inversions		Duplications		Translocations		Intrachromoso-mal rearrangements		Total	
	SVs	Somatic SVs	SVs	Somatic SVs	SVs	Somatic SVs	SVs	Somatic SVs	SVs	Somatic SVs	SVs	Somatic SVs	SVs	Somatic SVs
**EMM1**	4426	22	1748	48	53	4	79	1	1	1	8	8	6315	84
**EMM2**	4433	30	1712	62	68	1	49	0	0	0	6	6	6268	99
**EMM3**	4493	20	1694	41	44	2	53	5	4	4	11	11	6299	83
**EMM4**	4550	16	1755	36	54	3	74	3	0	0	2	2	6435	60
**Median (EMM)**	4463	**21**	1730	**45**	54	**3**	64	**2**	1	**1**	7	**7**	6307	**84**
**MM1**	4356	24	1597	32	61	2	52	13	9	9	0	0	6075	80
**MM2**	4528	20	1700	43	73	5	71	2	2	2	24	24	6398	96
**MM3**	4418	18	1655	24	64	1	63	3	2	2	0	0	6202	48
**MM4**	4296	17	1635	43	52	4	54	2	1	1	3	3	6041	70
**MM5**	4476	18	1743	46	75	9	51	9	4	4	8	8	6357	94
**MM6**	4268	11	1583	34	64	3	48	1	5	5	6	6	5974	60
**MM7**	4515	10	1710	26	62	1	66	5	5	5	1	1	6359	48
**Median (MM)**	4418	**18**	1655	**34**	64	**3**	54	**3**	4	**4**	3	**3**	6202	**70**

Table S6 Interchromosomal translocations detected in EMM and MM by optical mapping.

ID	Translocation	Chromosome A		Chromosome B		Affected gene(s)	Putative gene fusions	VAF (%)	* Function of affected genes
		Nr.	RefStart	Nr.	RefEnd				
**EMM1**	t(3;6)	3	112246734	6	87828316	*SLC9C1, AY927641*	*SLC9C1-AY927641*	39	-
**EMM3**	t(4;7)	4	104766887	7	69759315	*AUTS2*	-	12	A, E
	t(8;19)	8	17288095	19	14695184	*VPS37A, ZNF333*	*VPS37A-ZNF333*	8	A
	t(11;21)	11	89237343	21	40292426	*TYR, DSCAM*	*TYR–DSCAM*	8	A, C
	t(11;21)	11	12466990	21	40459650	*PARVA, DSCAM*	*PARVA-DSCAM*	12	A, C
**MM1**	t(2;8;22)	2	232918189	8	140735071	*NGEF, PTK2*	*NGEF-PTK2*	14	A, B, C, D, E
		2	231558893	22	23509455	-	-		-
		8	128139663	22	22240357	*IGL*	-		A, B
	t(3;11)	3	182852558	11	64942474	*ATP11B, C11orf85*	*ATP11B-C11orf85*	13	-
	t(3;12)	3	184270968	12	123617534	*ECE2, DDX55*	*ECE2-DDX55*	23	C
	t(6;8;17)	6	8032751	8	128014884	*BLOC1S5, PVT1*	*BLOC1S5-PVT1*	37	A, C, D
		6	7853269	17	76212325	*BMP6, RNF157*	*BMP6-RNF157*		A, B, C, D, E
		8	128025785	17	76093070	*PVT1, EXOC7*	*PVT1-EXOC7*		A, C, D
	t(8;22)	8	127358830	22	30936299	*CASC8, MORC2*	*CASC8 -MORC2*	18	A
**MM2**	t(3;19)	3	108219067	19	16907704	*IFT57, CPAMD8*	*IFT57-CPAMD8*	13	C
	t(11;14)	11	8469481	14	102111140	*STK33, HSP90AA1*	*STK33-HSP90AA1*	5	A, B, C, D, E
**MM3**	t(1;19)	1	38291095	19	16838518	-	-	15	-
	t(1;19)	1	39900616	19	15299192	*MYCL, BRD4*	*MYCL-BRD4*	18	A, B, C, D, E
	t(11;14)	11	70082628	14	104956159	*ANO1, AHNAK2*	*ANO1-AHNAK2*	6	A, C
**MM4**	t(4;14)	4	1883975	14	105896979	*WHSC1*	*WHSC1-IGH*	15	A, B, C, E
**MM5**	t(1;20)	1	170346977	20	53385582	*TSHZ2*	-	12	A
	t(6;8,17)	6	63634604	17	14986610	-	-	32	-
		8	34642485	17	17138691	*MPRIP*	-		A
	t(8;18)	8	35210482	18	31168889	-	-	13	-
**MM6**	t(2;5)	2	43890771	5	24087518	*LRPPRC, C5orf17*	*LRPPRC-C5orf17*	19	A, C
	t(2;8)	2	51663792	8	42093077	-	-	9	-

	t(3;4)	3	175027581	4	92382372	*NAALADL2, GRID2*	*NAALADL2-GRID2*	9	C
	t(4;5)	4	133789771	5	24087518	*C5orf17*	-	19	-
	t(9;13)	9	133789771	13	24087518	-	-	18	-
**MM7**	t(3;7)	3	52857383	7	82222486	*TMEM110, CACNA2D1*	*TMEM110-CACNA2D1*	5	A
	t(3;13;15)	3	122150053	13	77609535	*CASR, SCEL*	*CASR-SCEL*	7	A, C, D, E
		3	121362374	15	62582118	*STXBP5L, TLN2*	*STXBP5L-TLN2*		A, E
	t(6;15)	6	90834960	15	74561767	*ARID3B*	-	5	A, C
	t(13;16)	13	78285339	16	29813318	*RNF219-AS1, PRRT2*	*RNF219-AS1-PRRT2*	7	A, C

*****The function of affected genes is based on the data from NCBI gene database. Following gene panels were used: A: Cancer, B: MM, C: Cell cycle, D: Bone metabolism, E: Inflammation

Table S7 SVs on chromosome 1 in enrolled MM and EMM patients.

Patient ID	SV type	SV size (kbp)	Cytobands	Chromosome A		Chromosome B		VAF (%)	Affected genes	Gene panels				
				Nr.	RefStart	Nr.	RefEnd			Cancer	MM	Cell cycle	Bone metabolism	Inflammation
**EMM1**	intra-chrom	14502	1p36.33-p36.13	1	1451742	1	16010418	8	230	83	4	82	21	38
	deletion	12.3	1p32.3	1	54437326	1	54468426	13	0	0	0	0	0	0
	deletion	0.7	1p31.1	1	73986848	1	73989906	49	0	0	0	0	0	0
	deletion	0.7	1q21.2	1	149255041	1	149297922	12	0	0	0	0	0	0
	deletion	83.9	1q31.1	1	188862967	1	188948998	13	0	0	0	0	0	0
	deletion	0.9	1q41	1	219227885	1	219230864	69	0	0	0	0	0	0
**EMM2**	intra-chrom	46876	1p35.1-p31.1	1	33649122	1	80528558	13	475	157	9	174	23	64
	deletion	1611	1p32.3	1	52146402	1	53764220	6	22	7	0	8	4	2
	deletion	20.0	1p32.2	1	56859220	1	56897293	5	1	0	0	0	0	0
	deletion	311	1p32.2	1	57042204	1	57356850	6	1	0	0	0	0	0
	insertion	0.6	1p32.2	1	57981404	1	57987615	27	1	0	0	0	0	0
	intra-chrom	57947	1p32.1-p12	1	60494258	1	118466419	9	443	156	15	152	25	79
	deletion	14.5	1p31.1	1	70404062	1	70438713	20	1	0	0	0	0	0
	deletion	5.5	1p31.1	1	70766057	1	70784216	25	0	0	0	0	0	0
	intra-chrom	21501	1p22.2-p13.3	1	89446367	1	110953619	6	175	56	8	53	15	26
	deletion	7.3	1q25.3	1	182529994	1	182549190	61	1	0	0	0	0	0
**EMM3**	intra-chrom	7575	1p35.2-p34.3	1	30221701	1	37801246	15	120	42	1	46	6	14
	intra-chrom	7465	1p35.2-p34.3	1	30860870	1	38353947	8	126	44	1	47	6	15
	intra-chrom	12639	1p22.1-p21.1	1	93051297	1	105699124	9	81	18	4	19	5	7
	deletion	1.3	1p22.1	1	93329493	1	93335818	10	1	0	0	0	0	0
	intra-chrom	12839	1p21.1-p13.2	1	102272272	1	115119846	21	140	60	3	49	14	29
	deletion	5.8	1q42.3	1	235342739	1	235355292	18	1	0	0	0	0	0
**EMM4**	deletion	53.1	1p36.33	1	1679533	1	1743791	7	3	0	0	1	0	0
	insertion	2.5	1p36.12	1	21983384	1	22006562	13	2	0	0	0	0	0

	intra-chrom	36076	1p34.2-p31.1	1	39891936	1	75973164	7	363	117	9	131	21	51
	deletion	3687	1p34.2-p34.1	1	42633541	1	46350766	8	102	29	2	35	7	14
	intra-chrom	119844	1p34.1-1q23.3	1	44054030	1	163946030	8	1175	447	40	430	74	217
	insertion	2.5	1p13.2	1	111794239	1	111807945	11	1	0	0	0	0	0
	insertion	6.1	1p13.1	1	115530277	1	115543191	14	0	0	0	0	0	0
	deletion	0.8	1q31.3	1	195460336	1	195473816	16	0	0	0	0	0	0
	insertion	18.6	1q32.2	1	207515921	1	207534396	12	2	0	0	0	0	0
	insertion	6.0	1q32.3	1	213171761	1	213205644	12	1	0	0	0	0	0
**MM1**	insertion	5.9	1p36.33	1	1590522	1	1654114	17	7	2	1	1	0	1
	insertion	59.9	1q21.2	1	149365317	1	149390055	51	0	0	0	0	0	0
	insertion	10.9	1q21.3	1	152289954	1	152296885	13	0	0	0	0	0	0
	deletion	231.9	1q25.1	1	175947709	1	176185749	28	2	0	0	0	0	0
	deletion	3176	1q32.3-q41	1	214386812	1	217572960	21	8	3	0	4	2	1
	insertion	19.9	1q42.12	1	226337005	1	226338164	32	0	0	0	0	0	0
	deletion	315.8	1q43	1	238186340	1	238513554	23	1	1	0	0	0	0
	deletion	31.4	1q43	1	239651938	1	239694630	23	1	0	0	0	0	0
**MM2**	insertion	5.4	1p36.12	1	20372589	1	20396493	12	1	0	0	0	0	0
	deletion	14.6	1p31.1	1	83171695	1	83186312	19	0	0	0	0	0	0
	duplication	95.5	1q21.2	1	148669395	1	148764931	46	1	0	0	0	0	0
	insertion	2.4	1q23.2	1	161184787	1	161193876	13	1	0	0	0	0	0
	deletion	4.4	1q25.2	1	179360227	1	179368480	28	1	0	0	0	0	0
**MM3**	t(1;19)		1p34.3-19p13.11	1	38291095	19	16838518	15						
	t(1;19)		1p34.3-19p13.12	1	39900616	19	15299192	18						
	deletion	0.6	1q32.1	1	200212204	1	200225458	24	0	0	0	0	0	0
**MM4**	insertion	14.6	1p36.31	1	5999446	1	6006996	20	0	0	0	0	0	0
	insertion	1.8	1p12	1	119153160	1	119157652	27	0	0	0	0	0	0
	duplication	95.5	1q21.2	1	148669395	1	148764931	49	1	0	0	0	0	0
	insertion	2.4	1q23.3	1	161184787	1	161193876	28	1	0	0	0	0	0
**MM5**	insertion	23.0	1p36.13	1	16040685	1	16054506	9	0	0	0	0	0	0
	deletion	1.8	1p34.2	1	39074813	1	39085202	18	1	0	0	0	0	0

	deletion	5.4	1p31.3	1	62279900	1	62311886	20	1	0	0	0	0	0
	deletion	0.6	1p31.1	1	72996895	1	73015474	24	0	0	0	0	0	0
	duplication	79.6	1q24.2	1	168300624	1	168380191	60	2	1	0	0	0	0
	insertion	199.5	1q24.2	1	168300624	1	168380191	34	2	1	0	0	0	0
	t(1;20)		1q24.3-20q13.2	1	170346977	20	53385582	12						
	duplication	57.9	1q42.13	1	227147621	1	227205509	19	1	0	0	0	0	0
**MM6**	insertion	3.2	1p34.3	1	37899628	1	37903954	15	1	0	0	0	0	0
	deletion	14.6	1p31.1	1	83171695	1	83190595	17	0	0	0	0	0	0
**MM7**	insertion	5.7	1p36.33	1	1590522	1	1654114	5	0	0	0	0	0	0
	insertion	3.8	1p36.32	1	4070359	1	4096038	5	0	0	0	0	0	0
	deletion	23.2	1p36.12	1	21983384	1	22006562	17	2	0	0	0	0	0
	insertion	3.4	1p34.1	1	44413885	1	44419138	7	1	0	0	0	0	0
	duplication	47.2	1p21.3	1	99166139	1	99213371	7	0	0	0	0	0	0
	insertion	55.4	1p21.3	1	99180335	1	99199495	6	0	0	0	0	0	0
	deletion	721.6	1q21.1	1	143310164	1	144170341	25	9	1	0	0	0	0
	duplication	633.7	1q22-q23.1	1	156251622	1	156885360	5	28	11	2	12	0	7
	deletion	3.0	1q24.1	1	166768561	1	166777221	16	0	0	0	0	0	0
	deletion	0.7	1q32.1	1	200212204	1	200225458	15	0	0	0	0	0	0

Table S8 Intrachromosomal rearrangements across all chromosomes in MM and EMM samples.

Patient ID	Intra-chrom. rearrangements	SV size (kbp)	Cytobands	Chromosome A		Chromosome B		VAF (%)	Number of affected genes	Affected genes associations				
				Nr.	RefStart	Nr.	RefEnd			Cancer	MM	Cell cycle	Bone metabolism	Inflammation
**EMM1**	t(1;1)	14503	1p36.33-p36.13	1	1451742	1	16010418	8	231	84	4	84	22	38
	t(3;3)	20384	3q11.2-q13.31	3	94236905	3	114627463	46	137	47	6	36	4	16
	t(3;3)	6331	3q13.33-q21.3	3	120791064	3	127132678	31	72	25	3	24	4	15
	t(6;6)	5605	6q12-q14.1	6	62742306	6	68711990	22	11	4	0	2	0	1
	t(6;6)	16665	6q12-q14.1	6	62742306	6	79482533	22	60	21	1	2	3	9
	t(6;6)	15450	6q12-q14.1	6	64986937	6	80437318	6	61	20	1	18	3	8
	t(6;6)	11026	6q13-q14.1	6	68711990	6	79755701	23	50	18	1	16	3	8
	t(6;6)	6958	6q14.1-q14.3	6	80281222	6	87245378	16	31	13	0	9	3	3
**EMM2**	t(1;1)	57947	1p32.1-p12	1	60494258	1	118466419	9	444	156	15	152	25	79
	t(1;1)	46877	1p35.1-p31.1	1	33649122	1	80528558	13	475	157	9	174	23	64
	t(1;1)	21501	1p22.2-p13.3	1	89446367	1	110953619	6	174	56	8	53	14	26
	t(16;16)	24747	16p13.3-p12.1	16	2616774	16	27385578	7	305	89	11	99	12	44
	t(16;16)	24650	16p13.3-p12.1	16	3010544	16	27668650	17	287	83	12	85	12	42
	t(16;16)	24750	16p13.3-p12.1	16	2635753	16	27385578	8	305	89	11	89	12	43
**EMM3**	t(1;1)	7576	1p35.2-p34.3	1	30221701	1	37801246	15	121	42	1	46	6	14
	t(1;1)	7466	1p35.2-p34.3	1	30860870	1	38353947	8	126	44	1	47	6	15
	t(1;1)	12639	1p22.1-p21.1	1	93051297	1	105699124	9	81	18	4	19	5	7
	t(1;1)	12839	1p21.1-p13.2	1	102272272	1	115119846	21	140	60	3	33	14	29
	t(3;3)	27967	3q13.33-q25.1	3	121980403	3	149954119	8	242	98	6	84	14	39
	t(3;3)	27718	3q21.1-q25.1	3	122198317	3	149961776	13	239	96	5	83	13	37
	t(11;11)	34773	11p15.3-p11.2	11	12506422	11	47325290	8	226	101	6	90	22	40
	t(11;11)	30317	11p14.1-q12.1	11	27155667	11	57501781	10	233	68	6	69	17	30
	t(11;11)	22152	11q14.1-q22.3	11	79271831	11	101438780	9	105	43	0	35	7	11
	t(11;11)	43731	11p11.2-q14.2	11	44666666	11	88411497	14	703	263	15	239	54	112

	t(11;11)	22397	11q14.1-q22.3	11	79369242	11	101849155	10	107	43	0	35	8	12
**EMM4**	t(1;1)	36076	1p34.2-p31.1	1	39891936	1	75973164	7	363	117	9	131	21	51
	t(1;1)	119844	1p34.1-q23.3	1	44054030	1	163946030	8	1175	447	40	430	74	217
**MM2**	t(3;3)	14611	3q13.3-q21.1	3	108718151	3	123334502	18	136	60	7	38	5	24
	t(4;4)	5900	4p16.3-p16.1	4	4116398	4	10061592	22	79	17	0	19	3	9
	t(4;4)	36217	4p16.2-p14	4	4585745	4	40804114	27	198	64	2	63	26	28
	t(4;4)	10085	4p16.1-p15.31	4	10265563	4	20380667	23	40	18	1	14	12	7
	t(4;4)	37130	4p16.1-p12	4	10072592	4	47229162	23	153	56	3	57	28	26
	t(4;4)	35259	4p15.33-p12	4	12040957	4	47309662	19	146	52	3	55	24	24
	t(4;4)	21024	4p15.31-p14	4	19736783	4	40789951	10	86	29	1	33	10	14
	t(4;4)	26621	4p15.31-p12	4	20380667	4	47229162	23	111	35	2	42	15	18
	t(4;4)	10139	4q13.1-q13.3	4	64471748	4	74626024	15	82	46	4	36	9	29
	t(4;4)	10118	4q13.1-q13.3	4	64693043	4	74818434	22	83	47	4	37	9	29
	t(4;4)	20796	4q21.1-q22.3	4	75317695	4	96119255	25	125	57	6	53	15	28
	t(4;4)	22269	4q21.1-q23	4	75927442	4	98196108	22	118	53	5	48	15	27
	t(4;4)	6030	4q21.22-q22.1	4	82336357	4	88366632	14	48	24	4	24	8	11
	t(4;4)	18569	4q28.1-q31.21	4	122883408	4	141460111	20	62	19	2	19	10	8
	t(5;5)	43335	5q13.3-q23.1	5	76632808	5	119978410	16	209	78	7	70	12	35
	t(5;5)	50120	5q13.3-q23.2	5	77118925	5	127255688	19	232	79	7	71	13	33
	t(5;5)	10567	5q22.2-q23.2	5	112288925	5	122855774	24	59	17	0	13	3	8
	t(5;5)	9539	5q23.1-q23.3	5	119313747	5	128857700	10	42	11	0	7	2	3
	t(5;5)	14423	5q31.1-q32	5	132525483	5	146956548	5	233	80	6	81	17	46
	t(14;14)	23980	14q21.1-q23.3	14	40673049	14	64659156	5	162	66	3	52	12	22
	t(14;14)	29724	14q22.1-q31.1	14	51947074	14	81671064	10	281	115	4	101	22	41
	t(18;18)	28194	18q12.3-q22.2	18	41250664	18	69453185	18	151	54	5	54	8	31
	t(18;18)	27777	18q12.3-q22.2	18	42013880	18	69862559	13	151	55	5	54	8	31
	t(19;19)	6409	19p13.2-p13.11	19	10702479	19	17128307	13	209	74	7	73	19	45
**MM4**	t(11;11)	25365	11q14.1-q22.3	11	81669207	11	107054810	13	145	64	3	58	12	30
	t(14;14)	29271	14q22.1-q31.1	14	53235011	14	82510038	22	269	102	4	94	22	36
	t(22;22)	14140	22q11.23-q13.1	22	23818528	22	37963386	7	217	94	6	73	21	32

**MM5**	t(6;6)	12173	6q12-q14.1	6	68002113	6	80184361	61	58	20	1	18	3	8
	t(17;17)	10481	17p13.1-p11.2	17	7225163	17	17709763	37	188	71	6	66	15	34
	t(17;17)	7824	17p13.1-p11.2	17	8775856	17	16606473	84	78	22	1	22	3	12
	t(17;17)	5838	17p13.1-p11.2	17	10549727	17	16397226	54	54	15	0	14	3	8
	t(17;17)	6309	17p13.1-p11.2	17	10665585	17	16985890	86	67	19	2	15	3	11
	t(17;17)	8438	17p13.1-p11.2	17	10706191	17	19168519	36	116	38	3	29	9	16
	t(17;17)	7935	17p12-p11.2	17	14602052	17	22560688	45	133	42	4	35	9	16
	t(17;17)	7966	17p12-p11.2-1	17	14586278	17	22560688	38	133	42	4	35	9	16
**MM6**	t(6;6)	21508	6q25.1-q27	6	148464192	6	170116095	6	162	72	4	56	10	33
	t(6;6)	21305	6q25.1-q27	6	149056505	6	170369838	8	161	71	5	55	9	33
	t(13;13)	9463	13q13.3-q14.13	13	36453197	13	45917250	8	86	20	1	16	4	10
	t(13;13)	18934	13q14.11-q21.1	13	39970791	13	58907700	14	157	55	8	47	10	20
	t(13;13)	19975	13q14.11-q21.2	13	41555483	13	61536668	18	146	52	8	45	9	17
	t(13;13)	10157	13q21.33-q31.1	13	69990220	13	80154533	7	49	19	1	15	5	7
**MM7**	t(2;2)	113007	2p13.3-q32.1	2	70280948	2	183301119	7	749	274	20	252	37	108

Table S9 SVs within immunoglobulin *IGH/IGK/IGL* loci in MM and EMM patients.

SVs within the *IGH* (14q32.33; chr14:105,586,436-106,879,843), *IGK* (2p11.2; chr2:88,857,361-90,235,368), and *IGL* (22q11.2; chr222:22,026,076-22,922,913) loci together with translocations on immunoglobulin associated-chromosomes 2, 14, 22 in individual patients are presented.

Patient ID	IG locus	SV type	SV size (kbp)	Chromosome A		Chromosome B		VAF (%)
				Nr.	RefStart	Nr.	RefEnd	
**EMM1**	*IGH*	inversion	422	14	105906228	14	106327958	8
**EMM2**	*IGK*	deletion	27	2	90216214	2	90243376	21
**EMM3**	*IGK*	insertion	7	2	90216214	2	90223625	10
	IGL	deletion	32	22	22876286	22	22908320	13
**EMM4**	*IGL*	deletion	83	22	22176844	22	22260210	7
	IGL	insertion	34	22	22226007	22	22260210	5
**MM1**	*IGK*	t(2;8)		2	232918189	8	140735071	14
	IGK	t(2;22)		2	231558893	22	23509455	11
	IGL	t(8;22)		8	128139663	22	22240357	15
	IGH	duplication	1246	14	105082047	14	106327958	8
**MM2**	*IGH*	t(11;14)		11	8469481	14	102111140	5
**MM3**	*IGK*	deletion	60	2	88839699	2	88899440	24
	IGH	t(11;14)		11	69420345	14	106327958	15
**MM4**	*IGK*	deletion	37	2	89229705	2	89266833	20
	IGH	t(4;14)		4	1883975	14	105896979	15
**MM6**	*IGK*	t(2;5)		2	43890771	5	24087517	19
	IGK	t(2;8)		2	51663792	8	42093077	9
**MM7**	*IGK*	duplication	109	2	89900464	2	90009848	7
	IGH	duplication	823	14	105394788	14	106217512	5

VAF: Variant allele frequency.

Table S10 SVs and CNVs within *8q24/MYC* gene region detected by optical mapping, FISH and arrayCGH.

Patient ID	Cytogenetic analysis		Optical mapping							
	ArrayCGH	FISH	CNVs	SVs	SV size (kbp)	Chromosome A		Chromosome B		VAF (%)
						Nr.	RefStart	Nr.	RefEnd	
**EMM1**	normal	normal	normal	insertion	176	8	128321784	8	128323600	21
**EMM2**	normal	normal	normal	normal						
**EMM3**	loss	deletion*	deletion	deletion	2381	8	125261651	8	127716968	21
**EMM4**	normal	normal	normal	normal						
**MM1**	normal	normal	duplication	insertion	570	8	128011088	8	128014884	21
	normal	duplication*	duplication	duplication	223	8	128011088	8	128234073	13
	normal	normal	normal	t(6;8)		6	8032751	8	128014884	37
	normal	normal	normal	t(8;17)		8	128025785	17	76093070	43
	normal	normal	normal	t(8;22)		8	128139663	22	22240357	15
**MM2**	loss	normal	deletion	inversion	133975	8	126999605	8	129049253	10
**MM3**	normal	normal	normal	normal						
**MM4**	normal	normal	normal	normal						
**MM5**	normal	normal	normal	normal						
**MM6**	normal	normal	normal	normal						
**MM7**	normal	normal	normal	normal						

Table S11 CNVs on chromosome 1 in MM and EMM samples detected by optical mapping.

Patient ID	CNV type	CNV size (kbp)	Cytobands	Chromosome A		Chromosome B		CNV	Affected genes	Affected genes associations				
				Nr.	RefStart	Nr.	RefEnd			Cancer	MM	Cell cycle	Bone metabolism	Inflammation
**EMM1**	loss	4212	1p36.22-p36.21	1	11798011	1	16010418	1	82	19	1	18	2	9
	gain	103870	1q21.1-q44	1	144208666	1	248943333	3	1288	521	41	475	90	254
**EMM2**	loss	26416	1p35.11-p32.1	1	33649122	1	60065181	1	356	117	5	131	18	44
	loss	37908	1p31.1-p12	1	80528558	1	118456917	1	323	116	11	109	20	59
	gain	105664	1q21.1-q44	1	143278152	1	248943333	3	1297	522	41	475	90	254
**EMM3**	loss	3447	1p21.1	1	102272272	1	105719564	1	10	1	0	2	1	0
	loss	2516	1p13.2-p12	1	115119846	1	117635830	1	30	12	2	13	0	6
	loss	6181	1q43-q44	1	242762746	1	248943333	1	92	15	1	11	1	3
**EMM4**	loss	3893	1p36.32-p36.23	1	3864922	1	7757897	1	35	14	0	14	1	4
	loss	2926	1p36.22-p36.21	1	10456759	1	13382810	1	79	20	3	17	7	8
	loss	3300	1p34.2	1	39891936	1	43191819	1	50	16	1	18	3	8
	loss	2429	1p34.1-p33	1	44072317	1	46501513	1	59	15	0	17	3	7
	loss	45622	p31.1-p11.2	1	75978400	1	121608073	1	378	136	12	124	22	67
	loss	1952	1q21.1	1	143278152	1	145230161	1	20	3	0	1	0	1
	loss	7794	1q21.3-q23.1	1	151273576	1	159079122	1	265	111	10	105	14	53
**MM1**	gain	964	1q21.2	1	148140828	1	149105154	3	12	3	0	2	0	0
	gain	1496	1q23.2-q23.3	1	159913379	1	161409712	3	56	33	5	29	7	24
	loss	3223	1q32.3-q41	1	214344466	1	217567378	1	8	4	0	5	2	2
**MM2**	loss	35897	1p22.31-p11.2	1	85697789	1	121608073	1	328	120	12	106	21	57
	loss	2375	1q44	1	246568550	1	248943333	2	70	7	0	6	1	1
**MM3**	gain	39892	1p34.3-p34.2	1	38353947	1	39891936	3	25	9	0	11	0	5
**MM4**	loss	51148	1p31.1-p11.2	1	70460484	1	121608073	1	407	141	12	129	23	70
	gain	103362	1q21.1-q44	1	144228733	1	248943333	3	1288	521	41	475	90	254
**MM5**	gain	104485	1q21.1-q44	1	144092961	1	248943333	4	1288	521	41	475	90	254

**MM6**	gain	105665	1q21.1-q44	1	143278152	1	248943333	3	1297	522	41	475	90	254
**MM7**	loss	613	1q21.1	1	143523320	1	144136165	1	7	1	0	0	0	0
	gain	103232	1q21.1-q44	1	145439805	1	248943333	4	1275	516	41	474	90	253

Fig. S1 Intrachromosomal rearrangements (> 5Mb) within chromosome 1 in EMM patients.

Detection of deletion is supported by the copy number loss (CN=1) shown as red boxes above SVs. Large inversion (demonstrated, for example, in EMM3) inverts the orientation of the joined regions within the chromosome with no changes in copy number detected.

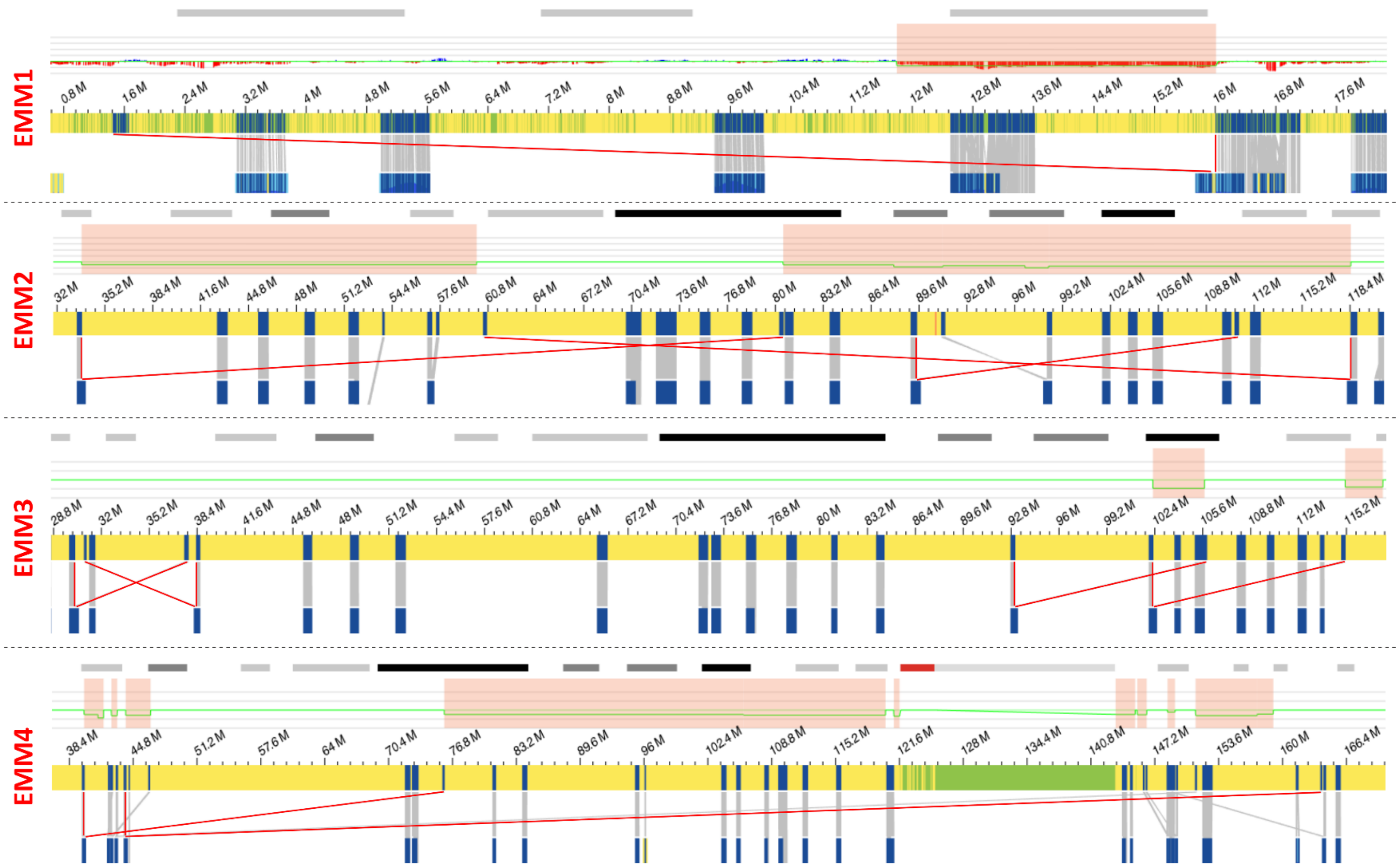

Fig. S2 Intrachromosomal rearrangements on chromosomes 4 and 14 – representative example.

(A) Large deletion accompanied by the copy number loss (CN=1, red part) in the whole affected region.

(B) Large inversion inside the chromosome 4 without change in copy number.

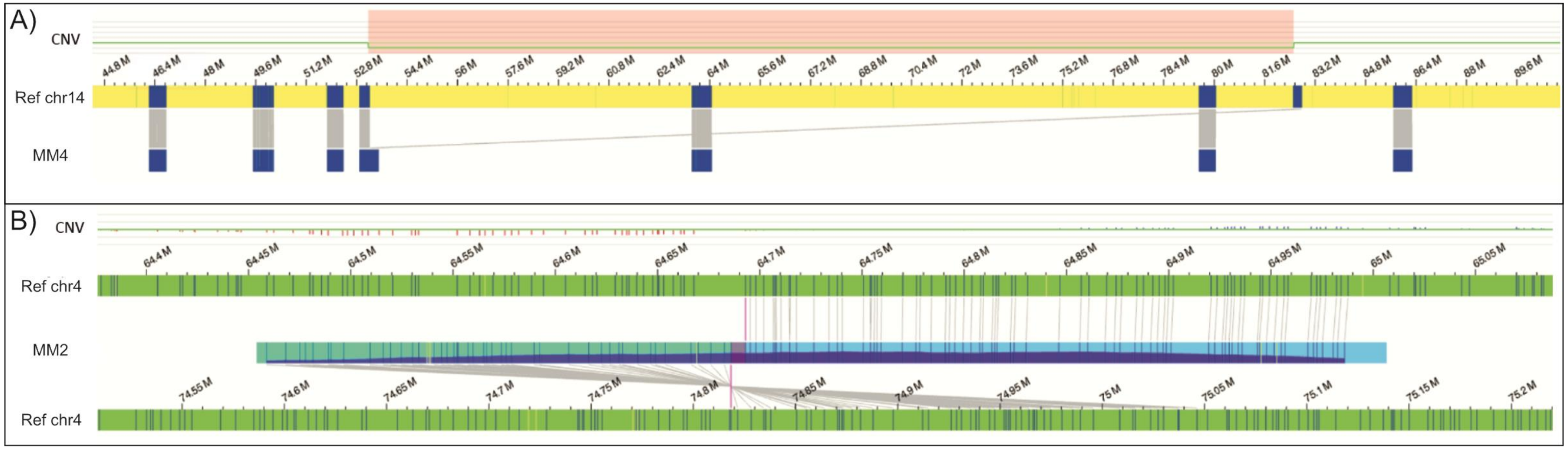

Fig. S3 Genomic aberrations within *IGH/IGK/IGL* immunoglobulin loci in EMM patients – representative examples.

(A) 422 kbp inversion within *IGH* gene loci.

(B) 83 kbp deletion within *IGL* gene loci.

(C) 7.4 kbp insertion within *IGK* gene loci.

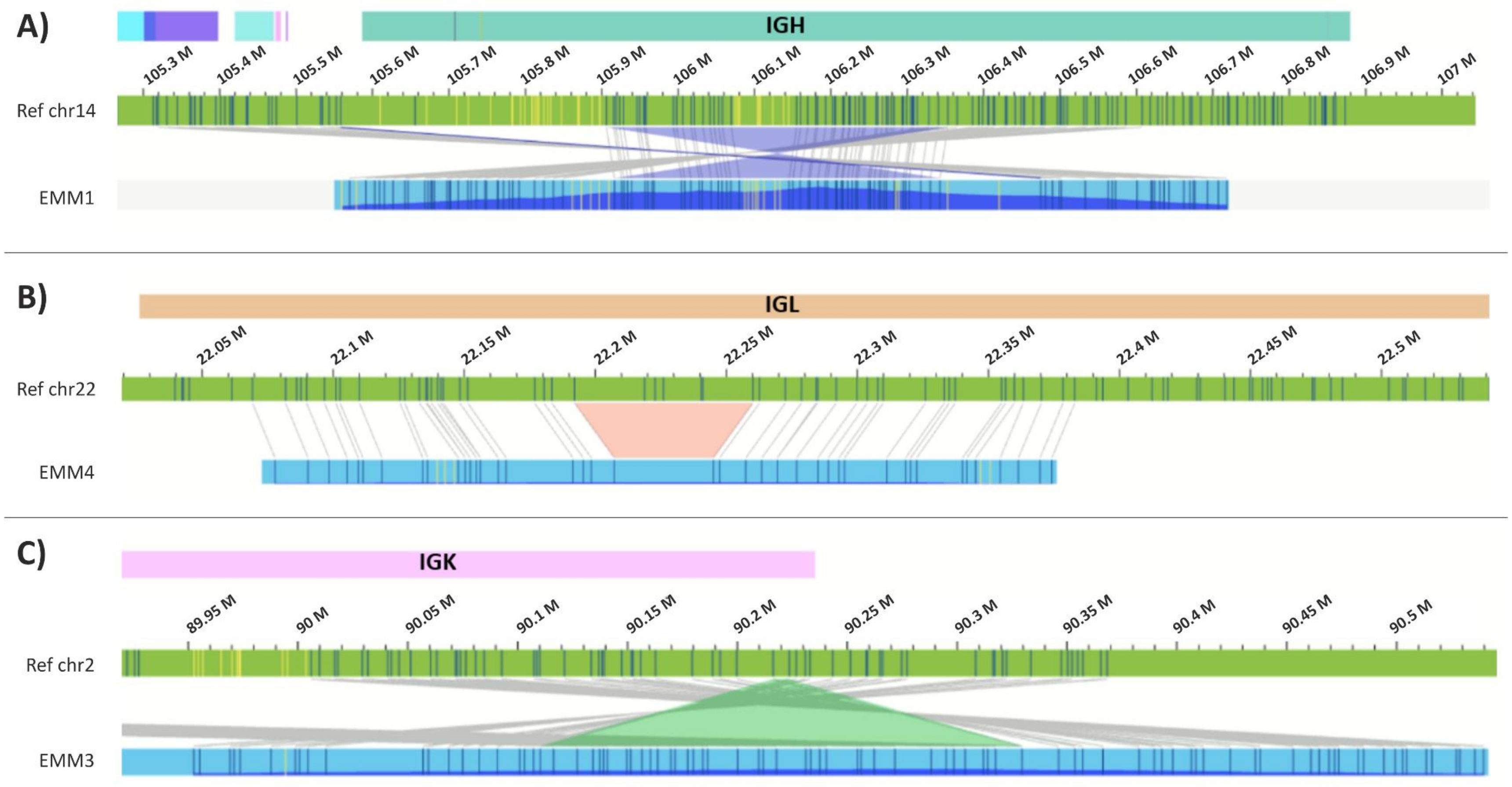

Supplement: Supplementary file 1 — Supplementary Information. [file 41598_2021_93835_MOESM1_ESM.pdf]
